# CoMetGeNe: mining conserved neighborhood patterns in metabolic and genomic contexts

**DOI:** 10.1186/s12859-018-2542-2

**Published:** 2019-01-10

**Authors:** Alexandra Zaharia, Bernard Labedan, Christine Froidevaux, Alain Denise

**Affiliations:** 1 Laboratoire de Recherche en Informatique (LRI), CNRS, Université Paris-Sud, Université Paris-Saclay, Orsay, 91405 France; 2 Institut de Biologie Intégrative de la Cellule (I2BC), CEA, CNRS, Université Paris-Sud, Université Paris-Saclay, Orsay, 91405 France

**Keywords:** Metabolic pathway, Gene neighborhood, Graph mining, Heterogeneous networks, Trail finding, Conserved interspecies patterns

## Abstract

**Background:**

In systems biology, there is an acute need for integrative approaches in heterogeneous network mining in order to exploit the continuous flux of genomic data. Simultaneous analysis of the metabolic pathways and genomic context of a given species leads to the identification of patterns consisting in reaction chains catalyzed by products of neighboring genes. Similar such patterns across several species can reveal their mode of conservation throughout the tree of life.

**Results:**

We present CoMetGeNe (*COnserved METabolic and GEnomic NEighborhoods*), a novel method that identifies metabolic and genomic patterns consisting in maximal trails of reactions being catalyzed by products of neighboring genes. Patterns determined by CoMetGeNe in one species are subsequently employed in order to reflect their degree of conservation across multiple prokaryotic species. These interspecies comparisons help to improve genome annotation and can reveal putative alternative metabolic routes as well as unexpected gene ordering occurrences.

**Conclusions:**

CoMetGeNe is an exploratory tool at both the genomic and the metabolic levels, leading to insights into the conservation of functionally related clusters of neighboring enzyme-coding genes. The open-source CoMetGeNe pipeline is freely available at https://cometgene.lri.fr.

**Electronic supplementary material:**

The online version of this article (10.1186/s12859-018-2542-2) contains supplementary material, which is available to authorized users.

## Background

Genomic data and chemical reactions embody the dual aspect of metabolism [1] that allows exploring the links between genome evolution and chemical evolution of enzyme-catalyzed reactions [2]. It is well-known that neighboring reactions corresponding to neighboring genes underline an evolutionary advantage in keeping the genes involved in succeeding reactions in close proximity [3, 4]. Finding almost identical sequences of reactions being catalyzed by products of neighboring genes in various species suggests that such sequences are made up of key enzymatic steps, best performed when their encoding genes are adjacent and co-transcribed. This type of metabolic and genomic organization strongly suggests the various species have been under strong evolutionary pressure to optimize the expression of enzyme-coding genes involved in successive reactions [5, 6].

In the present study, we focus on the identification of conserved metabolic and genomic patterns. Roughly speaking, metabolic and genomic patterns can be defined as corresponding neighborhoods of reactions and genes for a given species. *Conserved* metabolic and genomic patterns represent similar neighborhoods of reactions and genes for a variety of species. Interspecies comparisons based on conserved patterns may help to shed light onto the evolution of conserved metabolic and genomic neighborhoods. Differences in conserved patterns may signal various types of metabolism by pointing out alternative metabolic routes among several species. Such patterns may also suggest how metabolic maps may be completed by adding missing information derived from literature cross-checks. Furthermore, these patterns may uncover unexpected genomic organization motifs that are not self-evident but that nevertheless recurrently occur across several taxons.

The identification of metabolic and genomic patterns requires extraction of relevant information from metabolic and genomic contexts as well as its simultaneous integrated analysis. Knowledge extraction from biological networks has been the topic of numerous research efforts, mainly concentrated on ’omics’ data integration [7], network alignment and network mining. Network alignment has been used to align metabolic pathways [8, 9] and protein-protein interaction (PPI) networks [10, 11]. Network mining has multiple applications, such as prediction of RNA topology [12, 13] or identification of protein complexes in PPI networks [14].

The problem addressed in this paper involves knowledge extraction and processing from heterogeneous (as opposed to homogeneous) networks. Heterogeneous network sets present different types of information describing distinct aspects of related processes for the same biological entity. For example, a set of heterogeneous networks would include at least two items such as the genomic context of an organism and any one of the following networks: its metabolic pathways, its co-expression, co-regulation, and PPI networks.

The integrative analysis of several types of networks describing different processes for a given biological entity may lead to unexpected insights on the function of these processes, or on their respective relationships. Several early studies have thus concentrated on incorporating information from two heterogeneous networks. Ogata et al. [15] used EC numbers as the correspondence between reaction and gene networks in order to identify functionally related gene clusters. Enzyme Commission (EC) numbers represent a hierarchical classification system for enzymes, according to the chemical reactions that the enzymes catalyze [16]. Observing that enzymes encoded by genes belonging to an operon tend to catalyze successive reactions, Zheng et al. [17] developed a method for operon prediction using metabolic and genomic data. Spirin et al. [18] integrated metabolic networks and genomic associations in order to reveal evolutionary modules.

More recent works have proposed general frameworks for the integration of heterogeneous biological networks as either exact approaches [19–21] or heuristics [22, 23].

The pioneering approach of Boyer et al. [19] relied on the construction of an undirected correspondence multigraph representing the input networks and the relations between them. Common connected components were extracted from the correspondence multigraph in the form of syntons (neighboring genes for two or more species), metabolons (neighboring genes whose products are involved in connected metabolic reactions), and interactons (neighboring genes coding for physically interacting proteins). The same group further proposed a framework that handles larger numbers of input networks by building an undirected network alignment multigraph on-the-fly [20]. An improved method allowing the correspondence between aligned networks to be partial was employed for the detection of synteny blocks in bacteria [21].

In parallel, Bordron et al. [22] presented SIPPER, a method they illustrated on the integrated genomic and metabolic network of *Escherichia coli*. The integrated network is a directed weighted graph where each vertex is labeled with a reaction-gene pair. Arc weights in the integrated network represent the distance between genes within the genome. For any pair of reactions and a given *k*, SIPPER extracts subgraphs consisting of the *k* shortest paths between the source and destination reaction.

Fertin et al. [23] proposed a heuristic for determining a longest path *P* in a directed acyclic graph (DAG) such that *P* induces a connected subgraph in an undirected graph, where the two graphs have the same vertex set. The heuristic was used to find chains of reactions catalyzed by products of neighboring genes in one application, or by physically interacting proteins in another application. Since the heuristic can only be applied on DAGs, if the directed graph modeling a metabolic pathway contains cycles then a decomposition into DAGs is necessary [24]. Doing so is not straightforward and can lead to loss of solutions.

While interesting, the previously discussed frameworks have disadvantages related to the scope of our study. The extracted motifs are either subgraphs [19–22] or paths [23]. From a biological standpoint, it makes sense to allow for repeated vertices because metabolic pathways typically contain cycles. Hence, path extraction is not an appropriate option. We decided to focus on trail extraction, as trails can contain repeated vertices, but not repeated arcs [25]. In effect, a trail corresponds to a group of genes that are directly involved in a sequence of metabolic reactions. For example, the genes involved in the histidine operon encode successive steps in the biosynthesis of this amino acid; such successive steps form a trail. Another example (presented in the results section) is that of *mra* and *mur* genes involved in consecutive steps of peptidoglycan biosynthesis. For many bacterial species, the genes involved in these reactions are neighbors on the chromosome.

In this paper, we present CoMetGeNe (*COnserved METabolic and GEnomic NEighborhoods*), an exact method that identifies maximal trails of reactions being catalyzed by products of neighboring genes. CoMetGeNe allows for a flexible notion of neighborhood by defining parameters that authorize omitting a few reactions and/or adjacent genes. We subsequently employ CoMetGeNe for the identification of conserved metabolic and genomic patterns across a panel of 50 bacterial species representing the main phyla throughout the bacterial tree of life.

## Methods

### Model

**Fig. 1:**
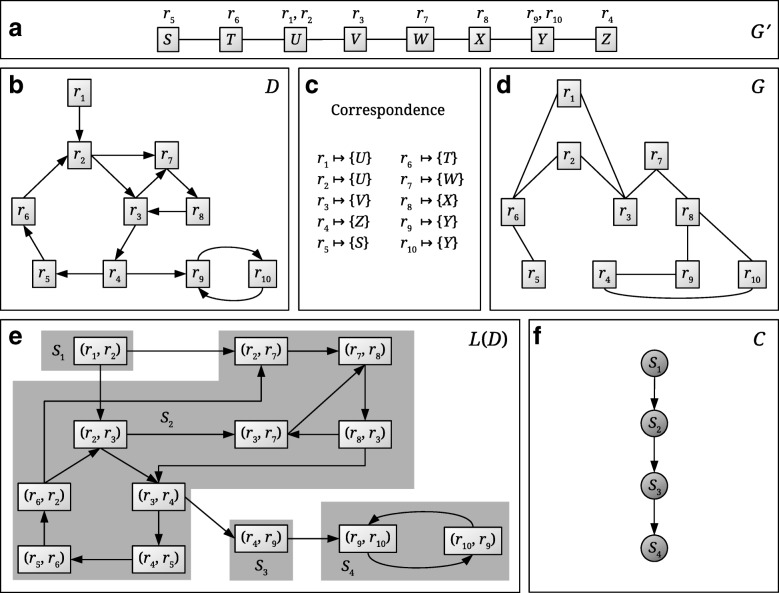
Schematic view of the CoMetGeNe model linking metabolic reactions and their encoding genes. **a** The undirected graph *G*^′^ represents the gene order of a given species. The reactions that gene products catalyze are indicated above each gene. **b** The directed graph *D* represents a metabolic pathway of the same species as in **a**. **c** The correspondence between reactions in *D* and genes in *G*^′^. **d** *G* is an undirected graph with the same vertex set as *D* built using the correspondence between reactions and genes. *G* represents gene neighborhood with respect to the reactions that the gene products catalyze. **e** *L*(*D*) is the line graph of *D*. By definition of the line graph, vertices of *L*(*D*) are arcs in *D*. Strongly connected components (SCCs) of *L*(*D*) are shaded in gray and assigned a label *S*_*i*_. **f** *C* is the condensation graph of *L*(*D*), obtained by replacing every SCC of *L*(*D*) with a single vertex
(ii) Metabolic pathways are represented as directed graphs with reactions for vertices (Fig. 1b). An arc leading from reaction *r*_*i*_ to *r*_*j*_ signifies that reaction *r*_*i*_ produces a metabolite that is a substrate for *r*_*j*_.
(iii) For a given species *S*, the relation between one of its metabolic pathways and its genome takes the form of a correspondence function associating genes to reactions: for any given reaction *r*, the correspondence function returns the set of genes of species *S* that code for enzymes catalyzing reaction *r* (Fig. 1c). This information can be found in a knowledge base such as KEGG (Kyoto Encyclopedia of Genes and Genomes) [26] which, for a given species, contains information on its metabolic pathways, the reactions that the species performs, and the genes associated to these reactions.

A non-spontaneous metabolic reaction is catalyzed by one or several enzymes. A given enzyme can be encoded by one or several genes. We regard metabolic pathways and genomic context as networks of reactions and genes, respectively. We represent the relation between metabolic pathways and their encoding genes using a classical model involving two graphs and a correspondence function:

(i) Genomes (viewed as gene networks) are represented as undirected graphs with protein-coding genes for vertices (Fig. 1a). Two protein-coding genes are connected by an edge if they are neighbors on the same strand of the same chromosome.

The method we propose, CoMetGeNe, requires two input graphs possessing the same vertex set. Thus, an additional undirected graph is constructed as described in [27] such that it reflects gene neighborhood with respect to the reactions that the gene products catalyze (Fig. 1d). The additional graph links two reactions *r*_*i*_ and *r*_*j*_ with an edge if at least one of the genes coding for an enzyme involved in reaction *r*_*i*_ is adjacent to a gene coding for an enzyme involved in *r*_*j*_. For example, genes *X* and *Y* are neighbors in *G*^′^ (Fig. 1a). Gene *X* codes for an enzyme involved in reaction *r*_8_, and gene *Y* codes for an enzyme involved in reactions *r*_9_ and *r*_10_. To reflect adjacency between genes *X* and *Y*, reactions *r*_8_ and *r*_9_, respectively *r*_8_ and *r*_10_, are linked by an edge in *G* (Fig. 1d).

### Finding metabolic and genomic patterns for a single species

#### Problem formulation

Given a metabolic pathway and the gene network for the same species, the objective is to identify a maximal number of consecutive reactions being catalyzed by products of neighboring genes. The problem was initially formulated under the name of LONGEST SUPPORTED PATH (LSP) [28], as follows:

LONGEST SUPPORTED PATH (LSP)

**Input:** A directed graph *D*=(*V*,*A*), an undirected graph *G*=(*V*,*E*).

**Output:** A longest path *P* in *D* such that *G*[*V*(*P*)] is connected.

In the above formulation, the notation *G*[*X*], where *G* is a graph and *X* is a set of vertices, stands for the subgraph of *G* induced by *X*, that is the subgraph of *G* with vertices of *X* as its vertex set, and where edges (or arcs in the directed case) are all the edges (or arcs) of *G* linking two vertices in *X* (see [29]). Thus the solution for LSP is a path in the directed graph *D* inducing a connected subgraph in the undirected graph *G*.

The vast majority of metabolic pathways, however, exhibit cycles (e.g. reversible reactions). Taking cycles into account requires that solutions be authorized to contain repeated vertices. Recall that, contrary to paths, trails can contain repeated vertices, but not repeated arcs [25].

We now define the concept of span and propose a new problem formulation that provides trails as solutions, instead of paths. The *span* of a trail *T* represents the number of distinct vertices in *T*. For example, if *T* is the trail (*r*_2_, *r*_3_, *r*_7_, *r*_8_, *r*_3_, *r*_4_) in Fig. 1b, then the span of *T* is 5, because vertex *r*_3_ is repeated.

MAXIMUM SPAN SUPPORTED TRAIL (MaSST)

**Input:** A directed graph *D*=(*V*,*A*), an undirected graph *G*=(*V*,*E*), an arc (*u*,*v*) in *D*.

**Output:** A trail of maximum span *T* in *D* passing through (*u*,*v*) such that *G*[*V*(*T*)] is connected.

Whereas LSP produces a path for every graph *D*, MaSST outputs trails of maximum span passing through arcs of *D* if the vertex sets of these trails induce connected subgraphs in *G*. The choice of producing a trail for every arc in *D* is deliberate in order to ensure that more than a single trail is retrieved per graph. For example, for graphs *D* (Fig. 1b) and *G* (Fig. 1d) and the arc (*r*_1_,*r*_2_), MaSST outputs one of the two following trails of span 8: (*r*_1_, *r*_2_, *r*_3_, *r*_7_, *r*_8_, *r*_3_, *r*_4_, *r*_9_, *r*_10_) or (*r*_1_, *r*_2_, *r*_7_, *r*_8_, *r*_3_, *r*_4_, *r*_9_, *r*_10_). For any other arc in *D*, the output of MaSST is either of the two following trails of span 9: (*r*_5_, *r*_6_, *r*_2_, *r*_3_, *r*_7_, *r*_8_, *r*_3_, *r*_4_, *r*_9_, *r*_10_) or (*r*_5_, *r*_6_, *r*_2_, *r*_7_, *r*_8_, *r*_3_, *r*_4_, *r*_9_, *r*_10_).

For practical purposes (see Path finding in the line graph below), we solve MaSST by using the line graph of *D*. Given a directed graph *D*, its line graph *L*(*D*) is a directed graph in which vertices are arcs in *D*. There is an arc in *L*(*D*) from a vertex *x* to another vertex *y* if and only if *x*=(*r*,*s*) and *y*=(*s*,*t*) with *r*, *s*, *t*∈*V*(*D*). For example, the graph in Fig. 1e is the line graph of the graph in Fig. 1b.

Let *D* be a directed graph and *L*(*D*) be its line graph. Let *P*=(*a*_1_, *a*_2_, …, *a*_*k*_) be a path in *L*(*D*), where *a*_*i*_=(*t*_*i*−1_, *t*_*i*_), 1≤*i*≤*k*, are arcs in *D*. The *trail in D corresponding to P*, denoted *L*^−1^(*P*), is the trail *T*=(*t*_0_, *t*_1_, *t*_2_, …, *t*_*k*−1_, *t*_*k*_). If *P* is an empty path, then *L*^−1^(*P*) is an empty trail. For example, if *P* is the path ((*r*_3_,*r*_7_), (*r*_7_,*r*_8_), (*r*_8_,*r*_3_)) in Fig. 1e, then *L*^−1^(*P*) is the trail (*r*_3_,*r*_7_,*r*_8_,*r*_3_) in the directed graph *D*.

We further propose MAXIMUM SPAN SUPPORTED CORRESPONDING TRAIL (MaSSCoT), a problem formulation equivalent to MaSST:

MAXIMUM SPAN SUPPORTED CORRESPONDING TRAIL (MaSSCoT)

**Input:** A directed graph *D*=(*V*,*A*), an undirected graph *G*=(*V*,*E*), an arc (*u*,*v*) in *D*.

**Output:** A path *P* in the line graph of *D* such that *L*^−1^(*P*) has maximum span, passes through (*u*,*v*), and *G*[*V*(*L*^−1^(*P*))] is connected.

Note that, as LSP has been shown to be NP-hard in the general case [23, 28], we have proved that MaSST and MaSSCoT are also NP-hard (Additional file 1).

#### Graph reduction

Fertin et al. [23] introduced the concept of a cover set of a path and proposed an algorithm to compute it. Briefly, given two graphs *D* (directed) and *G* (undirected) on the same vertex set *U*, as well as a path *P* in *D*, the cover set of *P* with respect to *D* and *G* is a maximal subset of *U* containing only vertices that might extend *P* into a path *P*^′^ such that *G*[*V*(*P*^′^)] and the undirected graph underlying *D*[*V*(*P*^′^)] stay connected. We have shown that, for a given arc (*u*,*v*) in *D*, reducing the input graphs *D* and *G* to the cover set *U*^′^ of (*u*,*v*) and feeding these reduced graphs *D*[*U*^′^] and *G*[*U*^′^] as input to MaSST and MaSSCoT yields the same solution as providing *D* and *G* as input (Additional file 2). In other words, graphs *D* and *G* are reduced to a strict minimum without loss of solutions.

#### Path finding in the line graph

The problem of trail enumeration in the directed graph *D* modeling a metabolic pathway is naturally solved by performing path enumeration in the line graph *L*(*D*). In other words, MaSST is solved using the MaSSCoT problem formulation. Path enumeration in *L*(*D*) is restricted to a minimum using the following three steps:

1. The strongly connected components (SCCs, see [29] for a definition) of *L*(*D*) and its condensation graph are computed, where a condensation graph results from replacing every SCC with a single vertex (Fig. 1e, f). Note that condensation graphs are acyclic by definition.
2. For every SCC of *L*(*D*), vertices acting as entry points from predecessor SCCs, as well as vertices acting as exit points to successor SCCs are determined. For example, in Fig. 1e, vertices (*r*_2_,*r*_3_) and (*r*_2_,*r*_7_) are entry points for SCC *S*_2_ when coming from the predecessor SCC *S*_1_. Vertex (*r*_3_,*r*_4_) in *S*_2_ is an exit point when heading to SCC *S*_3_. In *S*_3_, vertex (*r*_4_,*r*_9_) is both an entry point when coming from predecessor *S*_2_ and an exit point when heading to successor *S*_4_. *S*_1_ has no predecessor SCCs and *S*_4_ has no successor SCCs.
3. For every SCC *X* of *L*(*D*), path enumeration is performed only between strictly necessary source and destination vertices, as follows: (i) if *X* has at least one predecessor and one successor SCC, then paths are enumerated between feasible pairs of entry and exit points for these SCCs; (ii) if *X* has no predecessor and at least one successor SCC, then paths are enumerated between every vertex of *X* and exit points towards the successor SCC(s); (iii) if *X* has at least one predecessor and no successor SCC, then paths are enumerated between entry points from the predecessor SCC(s) and every vertex of *X*; (iv) only if *X* has no predecessor and no successor SCCs, paths are enumerated between every pair of vertices of *X*.

The paths obtained through step 3 above are evaluated in terms of span of their corresponding trails in *D* and the best candidate paths among them are retained. They are referred to as *best partial paths*.

#### Concatenation of partial paths

A path *Q* in the condensation graph *C* of *L*(*D*) is “translated” into one or several paths in *L*(*D*) by concatenating best partial paths in SCCs of *L*(*D*). Let *C*_*i*_ and *C*_*j*_ be two consecutive vertices of a path *Q* in *C* of length at least 1. Let *S*_*i*_ and *S*_*j*_ be the SCCs in *L*(*D*) corresponding to *C*_*i*_ and *C*_*j*_, respectively. Then *Q* has more than one corresponding path in *L*(*D*) if *S*_*i*_ has at least two exit points when heading to the successor SCC *S*_*j*_, or if *S*_*j*_ has at least two entry points when coming from the predecessor SCC *S*_*i*_.

For example, two paths in *L*(*D*) (Fig. 1e) correspond to path *Q*_1_ = (*S*_1_, *S*_2_, *S*_3_, *S*_4_) in *C* (Fig. 1f): *P*_1_=((*r*_1_,*r*_2_), (*r*_2_,*r*_7_), (*r*_7_,*r*_8_), (*r*_8_,*r*_3_), (*r*_3_,*r*_4_), (*r*_4_,*r*_9_), (*r*_9_,*r*_10_)) and $P^{\prime }_{1} = ((r_{1}, r_{2})$, (*r*_2_,*r*_3_), (*r*_3_,*r*_7_), (*r*_7_,*r*_8_), (*r*_8_,*r*_3_), (*r*_3_,*r*_4_), (*r*_4_,*r*_9_), (*r*_9_,*r*_10_)). The corresponding trails in *D* (Fig. 1b) are *L*^−1^(*P*_1_)=(*r*_1_, *r*_2_, *r*_7_, *r*_8_, *r*_3_, *r*_4_, *r*_9_, *r*_10_) and *L*^−1^(*P*1′)=(*r*_1_, *r*_2_, *r*_3_, *r*_7_, *r*_8_, *r*_3_, *r*_4_, *r*_9_, *r*_10_), both with span 8. Note that if *P*_1_ (respectively $P^{\prime }_{1}$) passed through arcs (*r*_4_,*r*_5_), (*r*_5_,*r*_6_), or (*r*_6_,*r*_2_), then *P*_1_ (respectively $P^{\prime }_{1}$) would be a trail instead of a path, which is not allowed.

In order to determine the solution to the MaSST problem, all paths in the condensation graph of *L*(*D*) are enumerated such that their corresponding paths in *L*(*D*) contain the SCC possessing the input arc (*u*,*v*) as vertex. If a path in *L*(*D*) obtained by concatenating best partial paths contains vertex (*u*,*v*), it is then evaluated in terms of its span by comparing it to the best current solution and by updating the current solution if necessary.

For example, let (*u*,*v*)=(*r*_2_,*r*_7_) (Fig. 1b). After translating path *Q*_1_ = (*S*_1_, *S*_2_, *S*_3_, *S*_4_) in *C* to a path in *L*(*D*), the best current solution *P*_1_ has span 8 as shown above. Now, suppose path *Q*_2_ = (*S*_2_, *S*_3_, *S*_4_) (Fig. 1f) is enumerated. There is one corresponding path in *L*(*D*) (Fig. 1e) passing through (*r*_2_,*r*_7_), obtained by concatenation of best partial paths in *S*_2_, *S*_3_, and *S*_4_. The best partial path in *S*_2_ ends in vertex (*r*_3_,*r*_4_) (which is an exit point when heading toward *S*_3_) and may start with any vertex in *S*_2_, provided the corresponding trail in *D* has maximum span. The path in *L*(*D*) corresponding to *Q*_2_ is therefore *P*_2_ = ((*r*_5_,*r*_6_), (*r*_6_,*r*_2_), (*r*_2_,*r*_7_), (*r*_7_,*r*_8_), (*r*_8_,*r*_3_), (*r*_3_,*r*_4_), (*r*_4_,*r*_9_), (*r*_9_,*r*_10_)), for which *L*^−1^(*P*_2_) has span 9. When *P*_1_ and *P*_2_ are compared, the best current solution now becomes *P*_2_ (because *L*^−1^(*P*_2_) has maximum span and because *G*[*V*(*L*^−1^(*P*_2_))] is connected (Fig. 1d)).

#### HNET algorithm

We propose HNET (*Heterogeneous NETwork mining*), an algorithm that solves the MaSST problem using the MaSSCoT formulation internally (Algorithm 1). Unlike the heuristic solution introduced in [23] to the LSP problem, HNET is an exact method. However, it is not exhaustive, meaning that if several trails of maximum span pass through a given arc (*u*,*v*) in *D*, then only one such trail is reported as solution. The bottleneck in HNET is path enumeration at line 5. In effect, the number of paths between two given vertices of a graph can be exponential with respect to the size of the graph. The exponential worst-case complexity of path enumeration is due to the NP-hardness of MaSST and MaSSCoT. The worst-case scenario occurs when all possible paths are enumerated between all pairs of vertices in a SCC. This scenario occurs in two distinct cases which nonetheless rarely arise in practice. The first case is that of SCCs of *D* that are completely disconnected from the rest of the graph. Sequences of reactions in metabolic pathways that are completely disconnected from the rest of the pathway are typically very short and therefore not limiting for exhaustive path enumeration. The second case is when *D* is strongly connected, corresponding to the infrequent situation in which a chain of reactions leads from any reaction *r*_*i*_ to any other reaction *r*_*j*_ of a given metabolic pathway, and vice versa.

In the following, assume: *D*=(*V*,*A*) is a directed graph; (*u*,*v*), an arc in *D*; *G*=(*V*,*E*), an undirected graph; *L*(*D*), the line graph of *D*; and *C*, the condensation graph of *L*(*D*).


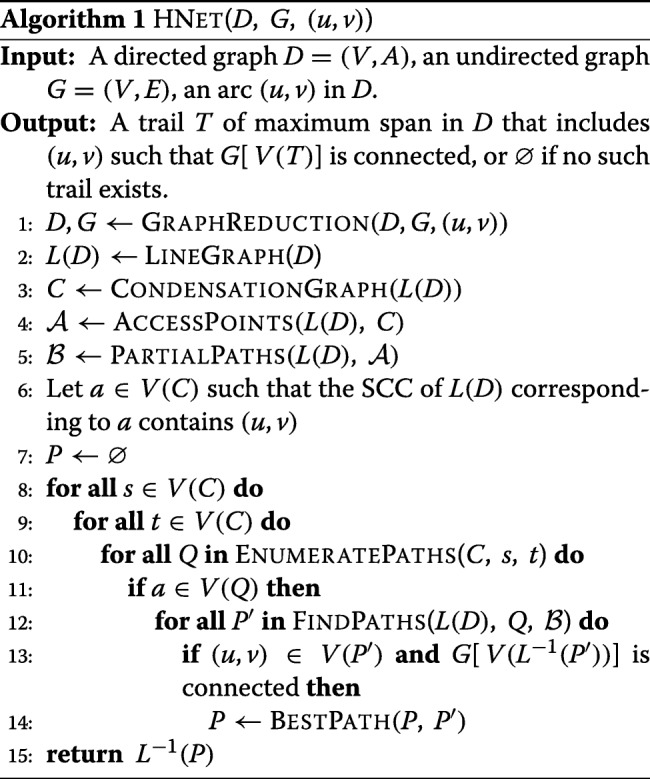


Algorithm GRAPHREDUCTION (line 1) returns the reduced graphs *D* and *G* (see Graph reduction above). For graphs *D* and *G* in Fig. 1 (panels b and d), the reduced and unreduced graphs are the same. LINEGRAPH (line 2) returns the line graph *L*(*D*) of the reduced input graph (Fig. 1b, e). CONDENSATIONGRAPH (line 3) returns the condensation graph of *L*(*D*), i.e. the directed acyclic graph obtained by replacing every SCC of *L*(*D*) by a single vertex (Fig. 1e, f).

Algorithm ACCESSPOINTS determines entry and exit points for every SCC *X* of *L*(*D*), from SCCs that are predecessors of *X* and toward SCCs that are successors of *X* (see Path finding in the line graph above, step 2). This information is stored in a data structure $\mathcal {A}$ that the algorithm returns at line 4. Algorithm PARTIALPATHS then uses $\mathcal {A}$ to compute best paths in every SCC *X* of *L*(*D*) (in terms of span of their corresponding trails in *D*) between all feasible pairs of source and destination vertices. Source vertices are entry points from predecessor SCCs if *X* has predecessors, and vertices of *X* otherwise. Reciprocally, destination vertices are exit points to successor SCCs if *X* has successors, and vertices of *X* otherwise. These paths, termed *best partial paths*, are stored in a data structure $\mathcal {B}$ that the algorithm returns at line 5 (see Path finding in the line graph above, step 3).

At line 6, HNET determines *a*, the vertex of *C* whose corresponding SCC in *L*(*D*) contains the input arc (*u*,*v*) as a vertex. Next, all possible paths in *C* are enumerated (lines 8-14) and, if they contain vertex *a*, the corresponding paths in *L*(*D*) are obtained by concatenation of best partial paths stored in $\mathcal {B}$. The best current solution is updated accordingly. A path *P* in *L*(*D*) qualifies as a best current solution if the trail in *D* corresponding to *P*, *L*^−1^(*P*), fulfills the following conditions: (i) it contains the input arc (*u*,*v*); (ii) it induces a connected subgraph in *G*; (iii) it has maximum span so far.

Algorithm ENUMERATEPATHS at line 10 returns all paths starting with vertex *s* and ending in vertex *t* in the condensation graph. If *s* and *t* are the same vertex, the algorithm returns either one. Algorithm FINDPATHS at line 12 returns all paths in *L*(*D*) corresponding to path *Q* in the condensation graph *C*, obtained by concatenation of best partial paths stored in $\mathcal {B}$. Given two paths in *L*(*D*), algorithm BESTPATH at line 14 returns the best current path, i.e. the path among the two whose corresponding trail in *D* has greater span than the other (see Concatenation of partial paths above).

Finally, HNET returns the trail in *D* corresponding to the best solution (line 15), effectively solving the MaSST problem. An additional consistency check is performed as detailed in [27] to ensure that the trail *L*^−1^(*P*) also “makes sense” when passing from *G* to the initial graph *G*^′^ (see Model and Fig. 1, panels a through d). It is checked whether vertices in *G*^′^ corresponding to the vertex set of the trail are connected. Note that [27] describes a heuristic solution to LSP (see Problem formulation).

**Fig. 2:**
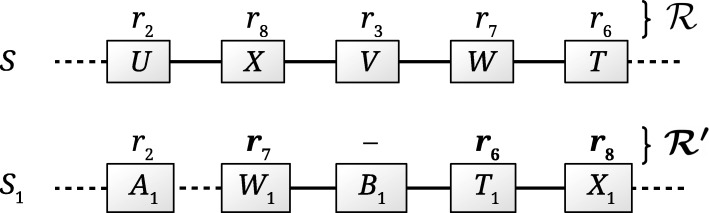
Gene neighborhood for species *S* and *S*_1_.

#### Allowing for skipped vertices

The MaSST and MaSSCoT formulations imply that solutions consist of strictly neighboring genes and reactions. As in a previous graph-based approach for the integration of heterogeneous biological data in another context [19], a preprocessing step was added to CoMetGeNe in order to allow for non contiguous reactions and/or genes. The preprocessing step consists in modifying the input graphs by adding arcs (respectively edges) between vertices separated by at most *δ*_*D*_ other reactions (respectively *δ*_*G*_ other genes). *δ*_*D*_ and *δ*_*G*_ are referred to as the *gap parameters*. Their value should be set quite low (e.g. at most 3) for ensuring that CoMetGeNe results are relevant from a biological point of view.

### Finding conserved metabolic and genomic patterns across multiple species

Here we show how trail finding, presented in the previous section, can be used to identify conserved interspecies metabolic and genomic patterns. We developed two methods for grouping trails obtained using the CoMetGeNe pipeline. They rely on examining trails of a given species, the *reference species*, in terms of either reactions or genes involved in these reactions, with the aim of comparing trails of the reference species with similar trails found for the remaining species. Both methods start out by pooling together all trails produced by the CoMetGeNe pipeline, for every species, every metabolic pathway, and every combination of the gap parameters.

For reasons explained below, both trail grouping methods were designed to treat trails as *reaction sets*, meaning that the order of reactions is not taken into account and that repeated reactions are ignored. In Fig. 1b, trails *t*_1_=(*r*_2_, *r*_7_, *r*_8_, *r*_3_, *r*_4_) and *t*_2_=(*r*_2_, *r*_3_, *r*_7_, *r*_8_, *r*_3_, *r*_4_) both have the same corresponding reaction set {*r*_2_, *r*_3_, *r*_4_, *r*_7_, *r*_8_}.

As previously explained, CoMetGeNe determines trails of reactions being catalyzed by neighboring genes. The definition of conserved patterns (in terms of metabolic and gene neighborhoods) needs to be able to accommodate slight variations between species. One such variation is encountering a different reaction order between trails. For example, if trails (*r*_9_,*r*_10_) and (*r*_10_,*r*_9_) are identified for two different species for the pathway in Fig. 1b, these trails naturally constitute a conserved pattern for the two species. Another variation that needs to be taken into account is best illustrated with the example of trails *t*_1_ and *t*_2_ above. If these trails are obtained for different species, the common feature is that both species perform the same five reactions using products of neighboring genes, irrespective of reaction order and of whether reaction *r*_3_ is repeated. Another example of variation that should not prevent the identification of conserved patterns is related to reactions (or genes) that are present in trails of some, but not all, of the species. For example, suppose the trails *t*_3_=(*r*_2_, *r*_3_, *r*_7_) and *t*_4_=(*r*_3_, *r*_7_, *r*_8_) are identified for two different species for the pathway in Fig. 1b. The fact that reactions *r*_3_ and *r*_7_ are common to both trails and are catalyzed by products of neighboring genes for both species should be identified as a conserved pattern. The necessity of accommodating these types of trail variations explains the choice for processing trails as reaction sets during the present trail grouping step.

Let $\mathcal {P}$ be the panel of selected species under study. Species $S \in \mathcal {P}$ denotes the chosen reference species. Let *R*_*S*_ be the set of all reaction sets of *S*. Note that reaction sets in *R*_*S*_ are not disjoint. From a biological standpoint, *R*_*S*_ represents the pool of trails of the reference species produced by CoMetGeNe, viewed in terms of reaction sets.

In the following, two genes of a given species are said to be *neighboring* if they are separated by at most three other genes on the same strand of the same chromosome.

#### Trail grouping by reactions

Briefly, the method of grouping trails by reactions consists in grouping reactions of the reference species according to the reaction sets they belong to. This grouping method focuses more on metabolic rather than genomic conserved patterns.

Grouping trails by reactions for the reference species *S* consists in constructing a table $T_{S}^{r}$ where rows represent reactions in every reaction set of *S* and columns represent the remaining species in $\mathcal {P}$. Table $T_{S}^{r}$ reflects conserved metabolic patterns between the reference species and the rest of the panel through the three possible symbols that can be assigned to each cell. These symbols allow to easily distinguish which reactions of the reference species are not present in the other species (blanks), and which are catalyzed by products of neighboring (crosses) and non neighboring (dots) genes of the other species.

For example, for the trail *t*=(*r*_6_, *r*_2_, *r*_3_, *r*_7_, *r*_8_) in Fig. 1b and the gene neighborhood in Fig. 2 for the reference species *S* and another species *S*_1_, $T_{S}^{r}$ is represented by the first ($\mathcal {R}$) and fourth (*S*_1_ in $T_{S}^{r}$) columns in Table 1. Reaction *r*_3_ is not performed by species *S*_1_. Reactions *r*_6_, *r*_7_, and *r*_8_ are performed by neighboring genes of *S*_1_ (*T*_1_, *W*_1_, and *X*_1_, respectively), whereas reaction *r*_2_ involves the product of a distant gene.

**Table 1:** Trail grouping by reactions and by genes for the reference species *S* against species *S*_1_

$\mathcal {R}$	*S* genes ($\mathcal {G}$)	*S*_1_ genes ($\mathcal {H}$)	*S*_1_ in $T_{S}^{r}$	*S*_1_ in $T_{S}^{g}$
*r*_2_	*U*	*A*_1_	.	.
***r***_***8***_	***X***	***X***_***1***_	x	x
*r*_3_	*V*	−		.
***r***_***7***_	***W***	***W***_***1***_	x	x
***r***_***6***_	***T***	***T***_***1***_	x	x
${\mathcal {R}^{\prime }}$	**Neigh. (** ${\boldsymbol{\mathcal {G}}^{\prime }}$ **)**	**Neigh. (** ${\boldsymbol{\mathcal {H}}^{\prime }}$ **)**		

Trail grouping by reactions and by genes are represented by the fourth (*S*_1_ in $T_{S}^{r}$) and fifth (*S*_1_ in $T_{S}^{g}$) columns, respectively (see text for definitions of the $T_{S}^{r}$ and $T_{S}^{g}$ tables). Labels without parentheses in table headers and footers refer to $T_{S}^{r}$, whereas labels within parentheses refer to $T_{S}^{g}$. $\mathcal {R}$ refers to both $T_{S}^{r}$ and $T_{S}^{g}$. $\mathcal {R'}$ refers only to $T_{S}^{r}$. Entries in bold in columns $\mathcal {R}$, *S* genes, and *S*_1_ genes respectively designate $\mathcal {R'}$ and neighboring genes in *S* and *S*_1_ (see table footer) for $T_{S}^{r}$. For $T_{S}^{g}$, entries in bold in columns $\mathcal {G}$ and $\mathcal {H}$ designate $\mathcal {G'}$ and $\mathcal {H'}$, respectively (see table footer). $\mathcal {R}$ represents a reaction set of *S*. $\mathcal {G}$ represents the group of neighboring genes of *S* whose products catalyze the respective reactions in $\mathcal {R}$. Symbols in column *S*_1_ in $T_{S}^{r}$ represent conserved metabolic patterns between species *S* and *S*_1_ for reactions in $\mathcal {R}$. Symbols in column *S*_1_ in $T_{S}^{g}$ represent conserved genomic patterns between species *S* and *S*_1_ for genes in $\mathcal {G}$. Roughly speaking, $\mathcal {R'}$ designates a maximal subset of $\mathcal {R}$ such that genes of *S*_1_ involved in reactions in $\mathcal {R'}$ are neighbors; $\mathcal {H}$ designates genes in *S*_1_ involved in reactions in $\mathcal {R}$; $\mathcal {H'}$ designates neighboring genes in $\mathcal {H}$ involved in reactions in $\mathcal {R}$. $\mathcal {H'}$ maximizes the number of genes in $\mathcal {G'}$, where genes in $\mathcal {H'}$ and $\mathcal {G'} \subseteq \mathcal {G}$ are involved in the same reactions in $\mathcal {R}$ (see text for formal definitions)

Rows in table $T_{S}^{r}$ represent reactions in *R*_*S*_ and are ordered by reaction sets of *S*. Note that a given reaction performed by species *S* appears several times in $T_{S}^{r}$ if it belongs to several reaction sets. Columns represent the remaining species in $\mathcal {P}$ and are ordered according to evolutionary distance to *S*, such that species phylogenetically closer to *S* have lower column indexes than species phylogenetically distant from *S*.

Let $T_{S}^{r}[i, j]$ denote the cell in $T_{S}^{r}$ on row *i* and column *j*. Let *r*_*i*_ denote the reaction of species *S* corresponding to row *i* in $T_{S}^{r}$. Let *S*_1_ denote the species corresponding to column *j* in $T_{S}^{r}$. Let $\mathcal {R} \subseteq R_{S}$ denote the reaction set of species *S* to which reaction *r*_*i*_ belongs. For the example presented above, the reaction set of species *S* that is investigated is $\mathcal {R} = \{r_{2}$, *r*_3_, *r*_6_, *r*_7_, *r*_8_} (see the first column ($\mathcal {R}$) in Table 1).

Let $\mathcal {R'}$ denote a maximal subset of $\mathcal {R}$ such that the genes of *S*_1_ involved in $\mathcal {R'}$ are neighbors. For the above example, the subset $\mathcal {R'}$ is {*r*_6_, *r*_7_, *r*_8_} (see $\mathcal {R'}$, i.e. entries in bold in the first column ($\mathcal {R}$) in Table 1) because reactions in $\mathcal {R'}$ involve the neighboring genes *T*_1_, *W*_1_, and *X*_1_, respectively (even though gene *B*_1_ is skipped).

One of the following three symbols is assigned to each cell $T_{S}^{r}[i,j]$:

- a cross (x) if $r_{i} \in \mathcal {R'}$.
- a dot (.) if $r_{i} \in \mathcal {R} - \mathcal {R'}$ and *r*_*i*_ is performed by species *S*_1_.
- a blank if $r_{i} \in \mathcal {R} - \mathcal {R'}$ and *r*_*i*_ is not performed by species *S*_1_.

For the above example (see the fourth column (*S*_1_ in $T_{S}^{r}$) in Table 1), the cells corresponding to reactions in $\mathcal {R'}$ receive a cross symbol (x). Since reaction *r*_2_ is performed in *S*_1_ by gene *A*_1_ which is not a neighbor of *W*_1_, *T*_1_, or *X*_1_, the corresponding cell on column *S*_1_ in $T_{S}^{r}$ receives a dot symbol (.). Finally, reaction *r*_3_ is absent from *S*_1_, therefore the corresponding cell receives a blank. The interpretation is that reactions *r*_6_, *r*_7_, and *r*_8_ are performed in species *S*_1_ by products of neighboring genes. Reaction *r*_3_ is absent from *S*_1_, whereas the gene involved in *r*_2_ is not a neighbor of genes involved in reactions *r*_6_, *r*_7_, and *r*_8_.

#### Trail grouping by genes

Here, we group CoMetGeNe reaction sets according to the gene order of the reference species. This second grouping method focuses more on genomic rather than metabolic conserved patterns. Two genes coding for enzymes involved in the same metabolic reaction are referred to as *functionally similar genes*. Functionally similar genes in two species can be either analogues (products of convergent evolution) or homologues (products of divergent evolution).

Grouping trails by genes consists in constructing a table $T_{S}^{g}$ where rows represent genes of the reference species *S* involved in reaction sets shared by *S* and at least one other species in $\mathcal {P}$, and columns represent the remaining species in $\mathcal {P}$. Table $T_{S}^{g}$ reflects conserved genomic patterns between the reference species and the rest of the panel through the two possible symbols that can be assigned to each cell. These symbols allow to easily differentiate genes of *S* with neighboring (crosses) and non neighboring (dots) functionally similar genes in other species.

For example, for the trail *t*=(*r*_6_, *r*_2_, *r*_3_, *r*_7_, *r*_8_) in Fig. 1b and the gene neighborhood in Fig. 2 for the reference species *S* and another species *S*_1_, $T_{S}^{g}$ is represented by the second ($\mathcal {G}$) and fifth (*S*_1_ in $T_{S}^{g}$) columns in Table 1. Genes *X*_1_, *W*_1_, and *T*_1_ of *S*_1_ respectively have the neighboring functionally similar genes *X*, *W*, and *T* in the reference species *S*.

Let $R_{S_{1}}$ be the set of all reaction sets for species $S_{1} \in \mathcal {P} - \{S\}$. Let *R* be the set of reaction sets defined by:

$$R = R_{S} \cap \left(\bigcup\limits_{S_{1} \in \mathcal{P} - \{S\}} R_{S_{1}} \right) $$

Hence, *R* represents the set of reaction sets common to *S* and at least one other species in $\mathcal {P}$. Let *G*_*S*_ be the set of genes of the reference species *S* that are involved in reactions belonging to reaction sets of *R*. From a biological standpoint, *G*_*S*_ represents the pool of genes of the reference species coding for enzymes involved in reaction sets common to *S* and at least one other species in $\mathcal {P}$.

Rows in table $T_{S}^{g}$ represent genes from *G*_*S*_ and are ordered by chromosome and strand, according to the position of genes on the strand. Columns represent the remaining species in $\mathcal {P}$ and are ordered according to evolutionary distance to *S* (see Trail grouping by reactions).

Let *S*_1_ denote the species corresponding to column *j* in $T_{S}^{g}$. Let $\mathcal {G}$ be a subset of *G*_*S*_ such that genes in $\mathcal {G}$ are neighbors on the same strand and chromosome of *S*. For the example presented above, the gene group of species *S* that is investigated is $\mathcal {G} = \{U, X, V, W, T\}$ (see the second column ($\mathcal {G}$) in Table 1).

Let $\mathcal {R}$ be the set of reactions in all reaction sets in which the genes in $\mathcal {G}$ are involved. Formally, $\mathcal {R}$ is the set of all reactions *r* such that: (i) there exists a reaction set *h* of species *S* such that *r*∈*h*, and (ii) there exists a gene $g \in \mathcal {G}$ such that *g* is involved in *r*. In other words, given a group $\mathcal {G}$ of neighboring genes of *S*, $\mathcal {R}$ is the set of reactions in trails common to *S* and at least one other species in $\mathcal {P}$ such that reactions in $\mathcal {R}$ are catalyzed by products of genes in $\mathcal {G}$. For the above example, $\mathcal {R}$ is {*r*_2_, *r*_3_, *r*_6_, *r*_7_, *r*_8_} (see the first column ($\mathcal {R}$) in Table 1).

Let $\mathcal {H}$ be the set of genes of *S*_1_ involved in reactions in $\mathcal {R}$. That is, given $\mathcal {R}$, the genome for species *S*_1_, and the correspondence between reactions in $\mathcal {R}$ and genes of *S*_1_, $\mathcal {H}$ is the set of genes in *S*_1_ (along with their position on the chromosome) such that every gene in $\mathcal {H}$ is involved in at least one reaction in $\mathcal {R}$. For the above example, $\mathcal {H} = \{A_{1}, X_{1}, W_{1}, T_{1}\}$ (see the third column ($\mathcal {H}$) in Table 1).

Let $\mathcal {H'} \subseteq \mathcal {H}$ be neighboring genes in $\mathcal {H}$, and let $\mathcal {G'} \subseteq \mathcal {G}$ such that genes in $\mathcal {H'}$ and $\mathcal {G'}$ are involved in the same reactions in $\mathcal {R}$. $\mathcal {H'}$ is chosen such as to maximize $|\mathcal {G'}|$, i.e. the number of genes in $\mathcal {G}$ involved in the same reactions as neighboring genes in $\mathcal {H}$.

For the above example, gene *A*_1_ is not a neighbor of gene *W*_1_, therefore $\mathcal {H'}$ must be a strict subset of $\mathcal {H}$. There are several possible strict non empty subsets of $\mathcal {H}$ of neighboring genes, other than singletons: {*W*_1_,*T*_1_}, {*W*_1_,*X*_1_}, {*T*_1_,*X*_1_}, and {*W*_1_,*T*_1_,*X*_1_}. The subset of $\mathcal {H}$ that is of interest is $\mathcal {H'} = \{W_{1}, T_{1}, X_{1}\}$, as it maximizes the number of genes in $\mathcal {G}$ involved in reactions in $\mathcal {R}$; $\mathcal {G'}$ is thus {*X*,*W*,*T*} (see $\mathcal {H'}$ and $\mathcal {G'}$, i.e. entries in bold in the third ($\mathcal {H}$) and second ($\mathcal {G}$) columns, respectively, in Table 1). The genes in $\mathcal {H'}$ can be considered neighbors because only gene *B*_1_ needs to be skipped as it does not code for an enzyme. The subset of reactions in $\mathcal {R}$ catalyzed by genes in $\mathcal {H'}$ is therefore {*r*_6_, *r*_7_, *r*_8_}.

Let $T_{S}^{g}[i, j]$ denote the cell in $T_{S}^{g}$ on row *i* and column *j*, where *i* is the index in *G*_*S*_ of a gene *g*_*i*_ in $\mathcal {G}$. One of the following two symbols is assigned to each cell $T_{S}^{g}[i,j]$:

- a cross (x) if $g_{i} \in \mathcal {G'}$.
- a dot (.) if $g_{i} \in \mathcal {G} - \mathcal {G'}$.

For the above example, cells for genes *U* and *V* receive a dot symbol (.), whereas cells for genes *X*, *W*, and *T* receive a cross symbol (x) (see the second ($\mathcal {G}$) and fifth (*S*_1_ in $T_{S}^{g}$) columns in Table 1). The interpretation is that genes *X*, *W*, and *T* of the reference species are involved in reactions catalyzed by neighboring genes in species *S*_1_. Notice that from trail grouping by genes alone it is not possible to decide whether the reactions catalyzed by genes *U* and *V* are absent from *S*_1_ or performed by products of non neighboring genes. Trail grouping by genes assigns dot symbols to *S*_1_ for genes *U* and *V* of the reference species. However, trail grouping by reactions assigns a dot symbol to *r*_2_ and a blank to *r*_3_, thus effectively distinguishing between reactions present in *S*_1_ (*r*_2_) and absent from *S*_1_ (*r*_3_).

### Pipeline

Trail finding (the HNET algorithm) and trail grouping are implemented in the form of CoMetGeNe, a Python 2.7 pipeline available under a MIT license at https://cometgene.lri.fr. CoMetGeNe is not compatible with Python 3. The following Python libraries are required: NetworkX (version ≥ 1.10 and ≤ 2.2) for graph handling, and lxml (version ≥ 3.5.0) for XML parsing. An Internet connection is mandatory for automatic data retrieval. Pipeline usage is detailed in Additional file 3.

#### CoMetGeNe files

CoMetGeNe automatically extracts the necessary data from KEGG using its REST API [30]. Metabolic pathways are stored in KGML format in a user-specified directory. Only pathways for primary and secondary metabolism excluding global and overview maps are extracted (i.e., maps whose KEGG identifier is at least 01100 are excluded). Genomic and EC number information are stored in binary format.

#### Trail finding

The script CoMetGeNe.py offers a convenient command-line interface for Finding metabolic and genomic patterns for a single species. The only required information is the species to be analyzed (designated by its three- or four-letter KEGG identifier [31]) and the directory where metabolic pathways of the species in question will be stored. Optionally, the gap parameters *δ*_*D*_ and *δ*_*G*_ can be specified (their default value being 0), as well as an output file for the results.

An important speedup is attained if CoMetGeNe is ran in parallel using the provided script CoMetGeNe_launcher.py. Restrictions inherent to KEGG limit pathway and genomic information retrieval to 3 and 2 threads, respectively. Trail finding in CoMetGeNe can, however, take full advantage of the maximum number of physical threads. See Results for CoMetGeNe run times.

A potential caveat when running CoMetGeNe in parallel is that KEGG may block concurrent downloads when using a fast Internet connection. Another potential caveat is that the machine may run out of memory on very large datasets (hundreds or thousands of species). In both cases, a possible workaround consists in adjusting parameters for CoMetGeNe_launcher.py. In the latter case, the dataset may be split into several smaller batches. For more details, see the “Trail finding” page on the CoMetGeNe website (https://cometgene.lri.fr/tfinding.html).

Storing metabolic pathways and genomic information for a given species allows CoMetGeNe to perform trail finding without re-downloading the same data for subsequent executions, e.g. when CoMetGeNe is ran for the same species but with different gap parameters.

CoMetGeNe uses a configurable timeout (defaulting to 5 min) for analyzing a given metabolic pathway. If the timeout is reached without producing any result, the pathway in question is “blacklisted” for the current species and set of gap parameters. This prevents CoMetGeNe from further attempting to analyze the given pathway for subsequent executions if the gap parameters increase. For example, a pathway that is blacklisted for (*δ*_*D*_=2, *δ*_*G*_=2) will not be further analyzed for (*δ*_*D*_,*δ*_*G*_)∈{(2,3),(3,2),(3,3)}. The blacklist is stored locally as a text file. Blacklisted pathways are computationally prohibitive due to the exponential number of enumerable paths. However, blacklisted pathways only amount to 3.3% of our dataset (121 out of 3709 pathways).

#### Trail grouping

Once CoMetGeNe results are available for several species, trail grouping can be performed in order to identify conserved metabolic and genomic patterns for several organisms (see Finding conserved metabolic and genomic patterns across multiple species). The script grouping.py provides this functionality and offers the possibility to save tables $T_{S}^{r}$ and $T_{S}^{g}$ in CSV format.

Three binary files are created when grouping trails by either reactions or genes. They contain pathway data, genomic information, and parsed CoMetGeNe results that can be reused when choosing another species as reference.

### Experimental setup

The test machine is a quad-core 2.6 GHz Intel Xeon E5-2623 v4 (Broadwell) with 10 MB L3 cache and 64 GB of RAM, running under Ubuntu GNU/Linux 16.04.3 LTS. Although the test machine has 64 GB of main memory, running CoMetGeNe on a single thread only requires approximately 100 MB of RAM.

## Results

Using CoMetGeNe, we performed trail finding and trail grouping on a panel of 50 bacterial species spanning major phyla of the bacterial tree of life (Table 2), with gap parameters *δ*_*D*_ and *δ*_*G*_ ranging from 0 to 3 (see Allowing for skipped vertices). Full results are available in Additional file 4. Genome size varies between 1062 and 8300 genes, with an average of 3269.5 genes. In total, 3709 pathways were extracted (74 pathways per species, on average). Metabolic and genomic data were extracted from KEGG on June 1, 2018 (see CoMetGeNe files and Additional file 5). See Additional file 6 for statistics per species on genome size, number and percentage of enzyme-coding genes, and number of pathways.

**Table 2:** The panel of 50 bacterial species chosen for this study

Species	Strain	Class	KEGG
			code
*Escherichia coli*	K-12 MG1655	*γ*-proteobacteria	eco
*Yersinia pestis*	CO92 (biovar Orientalis)	*γ*-proteobacteria	ype
*Vibrio cholerae*	O395	*γ*-proteobacteria	vco
*Shewanella putrefaciens*	CN-32	*γ*-proteobacteria	spc
*Pseudomonas aeruginosa*	PAO1	*γ*-proteobacteria	pae
*Xylella fastidiosa*	9a5c	*γ*-proteobacteria	xfa
*Ralstonia solanacearum*	GMI1000	*β*-proteobacteria	rso
*Neisseria meningitidis*	MC58 (serogroup B)	*β*-proteobacteria	nme
*Acidithiobacillus ferrivorans*	—	Acidithiobacillia	afi
*Agrobacterium radiobacter*	—	*α*-proteobacteria	ara
*Rickettsia rickettsii*	Iowa	*α*-proteobacteria	rrj
*Geobacter sulfurreducens*	PCA	*δ*-proteobacteria	gsu
*Nitrospira defluvii*	—	Nitrospira	nde
*Acidobacterium capsulatum*	—	Acidobacteriales	aca
*Desulfurispirillum indicum*	—	Chrysiogenetes	din
*Fusobacterium nucleatum*	subsp. *nucleatum* ATCC 25586	Fusobacteriia	fnu
*Denitrovibrio acetiphilus*	—	Deferribacteres	dap
*Thermodesulfatator indicus*	—	Thermodesulfobacteria	tid
*Aquifex aeolicus*	—	Aquificae	aae
*Bacillus subtilis*	subsp. *subtilis* 168	Bacilli	bsu
*Listeria monocytogenes*	EGD-e	Bacilli	lmo
*Staphylococcus aureus*	subsp. *aureus* N315 (MRSA/VSSA)	Bacilli	sau
*Lactobacillus acidophilus*	NCFM	Bacilli	lac
*Streptococcus pneumoniae*	ST556	Bacilli	snd
*Clostridium perfringens*	13	Clostridia	cpe
*Mycoplasma pneumoniae*	M129	Mollicutes	mpn
*Synechocystis sp.*	PCC 6803	Cyanobacteria (phylum)	syn
*Prochlorococcus marinus*	subsp. *marinus* CCMP1375	Cyanobacteria (phylum)	pma
*Chloroflexus aurantiacus*	—	Chloroflexia	cau
*Bifidobacterium breve*	ACS-071-V-Sch8b	Actinobacteria	bbv
*Corynebacterium glutamicum*	ATCC 13032 (Kyowa Hakko)	Actinobacteria	cgl
*Mycobacterium tuberculosis*	H37Rv	Actinobacteria	mtv
*Streptomyces coelicolor*	—	Actinobacteria	sco
*Deinococcus radiodurans*	—	Deinococci	dra
*Thermus thermophilus*	HB27	Thermi	tth
*Fimbriimonas ginsengisoli*	—	Fimbriimonadia	fgi
*Acetomicrobium mobile*	—	Synergistia	amo
*Thermotoga maritima*	MSB8	Thermotogae	tmm
*Caldisericum exile*	—	Caldisericia	cex
*Dictyoglomus thermophilum*	—	Dictyoglomia	dth
*Fibrobacter succinogenes*	—	Fibrobacteria	fsu
*Gemmatimonas aurantiaca*	—	Gemmatimonadetes	gau
*Chlorobium phaeobacteroides*	DSM 266	Chlorobia	cph
*Bacteroides fragilis*	YCH46	Bacteroidia	bfr
*Rhodopirellula baltica*	—	Planctomycetia	rba
*Chlamydia pneumoniae*	CWL029	Chlamydiia	cpn
*Opitutus terrae*	—	Opitutae	ote
*Borrelia burgdorferi*	N40	Spirochaetia	bbn
*Elusimicrobium minutum*	—	Elusimicrobia	emi
*Helicobacter pylori*	26695	*ε*-proteobacteria	heo

See Additional file 6 for statistics per species (genome size, number and percentage of enzyme-coding genes, number of pathways, number of trails, average and median trail span, number of trails of span between 1 and 3, between 4 and 10, and 11 or higher)

A total of 4179 CoMetGeNe trails were identified, of which 2620 (62.7%) occur solely in a single species. The number of trails per species varies between 19 and 501, with an average of 201 trails. Table 3 shows trail span distribution (recall that the span of a trail represents the number of distinct reactions in the trail). The majority of trails are short, consisting of up to three distinct reactions. Other trails, however, have as many as 35 unique reactions, e.g. for the fatty acid biosynthesis pathway in *Bifidobacterium breve* (bbv) and *Streptococcus pneumoniae* (snd), see Additional file 7 for the full list of reactions. See Additional file 6 for statistics per species on the number of trails, as well as the average and median trail span.

**Table 3:** Distribution of trail span

Trail span	Percentage of trails
1 −3	56.4%
4 −10	38.7%
11 −35	4.9%

The trail finding run time for CoMetGeNe for the whole dataset of 50 bacterial species (Table 2) was under 4 hours and 30 min when using 8 threads (see Additional file 8 for execution times per species). The trail finding run time does not take into account the time required to automatically retrieve data from KEGG, as this is dependent upon the Internet connection speed and upon the number and size of the selected genomes. In our experimental setup, metabolic pathways and genomic information were retrieved in 12 and 76 min, respectively. When each of the species in the dataset is taken in turn as reference species, trail grouping by reactions and by genes takes approximately one hour in total. Thus, data retrieval from KEGG for our bacterial panel (Table 2), followed by trail finding and trail grouping, amounted to approximately 7 hours.

Available software for detecting metabolic reactions being catalyzed by products of neighboring genes is scarce. The C3Part/Isofun package [32] implements the methods proposed in [19–21]. It takes as input a file in extended DIMACS format describing the layered multigraph [21], and outputs connected components common to both layers in the multigraph. Unlike C3Part/Isofun, the CoMetGeNe software does not require the user to prepare any input files as they are extracted automatically from KEGG, thus rendering CoMetGeNe extremely simple to use. In order to evaluate C3Part/Isofun, we constructed the input files corresponding to the pathways and genome of *Escherichia coli*. Since C3Part/Isofun produces undirected subgraphs whereas CoMetGeNe outputs trails, comparing the two programs is not straightforward. We therefore investigated whether the reaction sets corresponding to CoMetGeNe trails may be found among the results of C3Part/Isofun and vice versa. CoMetGeNe and C3Part/Isofun extracted 114 trails and 65 subgraphs, respectively. While most results are common to the two programs, CoMetGeNe detected 50 additional trails with respect to C3Part/Isofun, whereas C3Part/Isofun identified 7 additional subgraphs with respect to CoMetGeNe. These subgraphs, being undirected, do not translate actual metabolic routes, some of them corresponding to partly overlapping CoMetGeNe trails. The comparison between CoMetGeNe and C3Part/Isofun is detailed in Additional file 9.

CoMetGeNe recovered trails in most major pathways of *E. coli*, including nucleotide metabolism, fatty acid biosynthesis, carbohydrate metabolism, and amino acid metabolism. For example, CoMetGeNe trails were detected for the biosynthesis of every amino acid with the exception of tyrosine and tryptophan. In the following, two case studies in *E. coli* are illustrated, the first involved in glycan metabolism, and the second one in amino acid metabolism.

### Exploring steps of peptidoglycan biosynthesis

**Fig. 3:**
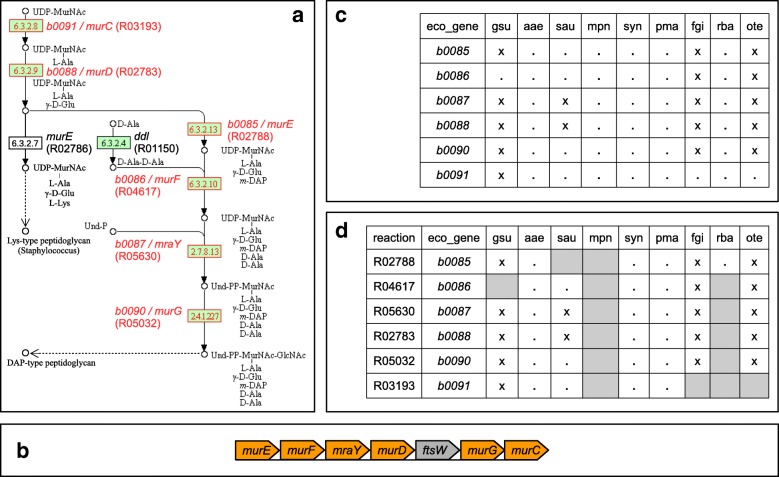
CoMetGeNe trail for *E. coli* in the peptidoglycan biosynthesis pathway. **a** Partial view of the peptidoglycan biosynthesis pathway, adapted from KEGG PATHWAY, map eco00550 (August 3, 2018 version). Green reaction nodes designate reactions present in *E. coli*. The gap parameter *δ*_*G*_ was set to one (thus allowing to skip one gene). Reactions in the trail (red contours) are labeled with the corresponding KEGG reaction identifiers (R numbers) and with the Blattner identifiers and gene names of genes coding for the respective enzymes. The genes involved in this trail are neighbors on the positive strand of the *E. coli* chromosome (see **b**). Dashed arrows from a metabolite *m* to another metabolite *m*^′^ signify that a chain of reactions, omitted in this figure for clarity, leads from *m* to *m*^′^. **b** Genomic context for genes involved in the trail in **a**. The gene in gray is not involved in the trail. **c** Group of homologous genes involved in the trail in **a**, present in various species. See Trail grouping by genes for details. **d** Group of reactions defining the trail in **a**. *eco_gene* designates the gene in *E. coli* whose product catalyzes the corresponding reaction. See Trail grouping by reactions for details. In this figure, all reactions that are absent from certain species (blanks) are highlighted in gray

Figure 3a illustrates trail finding by CoMetGeNe on the well-studied biological process of peptidoglycan biosynthesis. Peptidoglycan is the main constituent of the bacterial cell wall and is present in the vast majority of bacteria. The trail in Fig. 3a was recovered in the peptidoglycan biosynthesis pathway of *Escherichia coli* (eco00550) and represents the conversion of UDP-N-acetylmuramate (UDP-MurNAc) into a precursor of DAP-type peptidoglycan. Figure 3b shows the genes coding for enzymes involved in this trail: *murE* (*b0085*), *murF* (*b0086*), *mraY* (*b0087*), *murD* (*b0088*), *murG* (*b0090*), and *murC* (*b0091*). Note that the trail produced by CoMetGeNe was obtained by skipping gene *ftsW* (*b0089*), with gap parameter *δ*_*G*_ set to 1.

The skipped gene encodes the FtsW protein, which plays an essential role in cell division [33]. Moreover, it has been shown that FtsW is also a transporter of peptidoglycan precursors across the inner membrane [34]. It is therefore interesting that the gene encoding this transporter, although not included in the trail, is found in the same neighborhood as peptidoglycan biosynthesis genes. This underlines the capacity of CoMetGeNe to identify trails of reactions that are compatible with their genomic context.

Trail grouping was performed for *E. coli* (eco) as reference species. Figures 3c and 3d respectively illustrate the portions in tables $T^{g}_{\texttt {eco}}$ (trail grouping by genes) and $T^{r}_{\texttt {eco}}$ (trail grouping by reactions) corresponding to the trail in Fig. 3a, for *E. coli* and 9 other bacterial species presenting interesting features. Trail grouping by genes and by reactions for the full dataset is available in Additional files 10 and 11, respectively.

Trail grouping by genes identifies genes of the reference species with neighboring functionally similar genes in other species. The degree of conservation of gene neighborhood for the genes involved in a given trail is directly proportional to the number of cross symbols (x) in $T_{S}^{g}$ for the reference species *S*. The density of crosses in $T_{\texttt {eco}}^{g}$ (Additional file 10) confirms that the trail in Fig. 3a is frequently found for the species in the dataset, albeit with varying degrees of conservation of gene neighborhood. This finding represents a positive control, being consistent with the fact that most bacteria possess peptidoglycan cell walls.

Cells with dot symbols (.) in $T_{\texttt {eco}}^{g}$ (Fig. 3c and Additional file 10) do not allow to distinguish between non neighboring and missing genes. However, Fig. 3d identifies species with missing reactions (in gray in the figure) with respect to *E. coli*: *Geobacter sulfurreducens* (gsu), *Staphylococcus aureus* (sau), *Mycoplasma pneumoniae* (mpn), *Fimbriimonas ginsengisoli* (fgi), *Rhodopirellula baltica* (rba), and *Opitutus terrae* (ote). The remaining species perform all the reactions but do not necessarily have contiguous genes coding for the required enzymes. Of the six species with missing reactions with respect to *E. coli*, *M. pneumoniae* (mpn) is a negative control, as it is well-known that it is devoid of a cell wall [35].

*G. sulfurreducens* (gsu), a deltaproteobacterium [36] with a peptidoglycan dry weight fraction of 4% [37], is reportedly missing reaction R04617 (Fig. 3d) which should be catalyzed by MurF (Fig. 3a). However, the KEGG GENES entry *GSU3073* is annotated as *murF* [38] but the gene is not associated to reaction R04617 in the pathway map as of the writing of this paper (August 3, 2018 version of map gsu00550). *GSU3073* is located in the same gene neighborhood as the other genes encoding the enzymes for the reactions in Fig. 3d. Moreover, as revealed by CoMetGeNe, every other reaction in the trail is performed by enzymes encoded by neighboring genes. We confirmed the functional annotation *murF* for gene *GSU3073* by performing a protein BLAST [39] for the *E. coli* MurF query sequence against *G. sulfurreducens* (NCBI taxon 35554). The matching protein WP_010943698 (40% identity, 98% query cover, E-value 1e − 76) corresponds to gene *GSU3073* via the identical protein YP_006589581. The missing reaction R04617 for *G. sulfurreducens* (gsu) is hence an instance of incorrect annotation in the KEGG knowledge base in the sense that gene *GSU3073* has not been associated to reaction R04617.

*S. aureus* (sau) is a Gram-positive bacterium [40], well known to produce lysine-type peptidoglycan (dashed arrow in Fig. 3a) instead of DAP-type peptidoglycan. This is accomplished using the alternative route passing through reactions R02783 (EC 6.3.2.9) and R02786 (EC 6.3.2.7). The metabolic route leading to lysine-type peptidoglycan in *Staphylococcus* shares the two reactions catalyzed by MurC (R03193) and MurD (R02783) with the route leading to DAP-type peptidoglycan. Equivalents of the other four reactions in Fig. 3d exist in lysine-type peptidoglycan biosynthesis and are performed by the same enzymes (MurE, MurF, MraY, and MurG) on UDP-MurNAc substrates having lysine (instead of DAP) residues. As illustrated in Fig. 3d, only two genes among those involved in peptidoglycan biosynthesis in *S. aureus* are neighbors (*mraY* and *murD*).

*F. ginsengisoli* (fgi), a member of the recent Armatimonadetes phylum, is reportedly missing reaction R03193 (EC 6.3.2.8 in Fig. 3a) which should be catalyzed by MurC (Fig. 3d). Since this species has been described as synthesizing DAP-type peptidoglycan [41] and it also performs every other reaction in the trail in Fig. 3a using products of neighboring genes, we proceeded to a protein BLAST [39] search against *F. ginsengisoli* (NCBI taxon 1005039) with the MurC sequence of *Chthonomonas calidirosea*, another member of the Armatimonadetes phylum, as query. The search was inconclusive, as the best match (WP_025227986) corresponds to gene *OP10G_4783* which encodes a hypothetical protein roughly half the size of MurC and with no known domains, and the second best match (AIE88152) corresponds to gene *OP10G_4784* which is a D-alanine–D-alanine ligase (*ddl*), being involved in another reaction in the peptidoglycan biosynthesis pathway (see *ddl* in Fig. 3a). Intriguingly, *OP10G_4784* has been annotated as a UDP-N-acetylmuramate–L-alanine ligase, which describes the role of MurC. Furthermore, *OP10G_4784* has the additional *Mur_ligase_C* annotation, corresponding to the C-terminal Mur ligase domain, but MurC should possess additional middle and catalytic domains. Although the STRING database [42] reports that fusions of *murC* and *ddl* occur frequently in the Chlamydiae phylum, it does not appear to be the case for *OP10G_4784* due to missing Mur ligase domains and different sequence size with respect to *murC*–*ddl* fusions in Chlamydiae. Interestingly, a Mur ligase catalytic domain is reported for the short neighboring gene *OP10G_4785*, also annotated as a UDP-N-acetylmuramate–L-alanine ligase. A KEGG ortholog search for *OP10G_4785* reveals longer *murC* ortholog sequences in other species. Two hypotheses are therefore possible: (i) the activity EC 6.3.2.8 is performed jointly by products of genes *OP10G_4784* and *OP10G_4785* in *F. ginsengisoli* (fgi), or (ii) the open reading frame for *OP10G_4784* was incorrectly predicted, the *ddl* coding sequence erroneously including a *Mur_ligase_C* domain that may in fact belong to *OP10G_4785*.

*R. baltica* (rba), as other Planctomycetes, has been thought to be lacking peptidoglycan [43]. Consistent with annotations in KEGG reflecting the existing genome annotations, CoMetGeNe only identifies one reaction among the six in the trail in Fig. 3a as being present in *R. baltica*. In addition, no peptidoglycan biosynthesis genes are currently listed in the STRING database [42] for other Planctomycetes beside members of the *Planctomyces* genus. However, Jeske et al. [44] have biochemically demonstrated that sugar and peptide components of peptidoglycan are present in Planctomycetes. The study also uses an *in silico* approach to identify candidate peptidoglycan biosynthesis genes in *R. baltica* and other Planctomycetes. The fact that the findings of this study are yet to be reflected in existing annotations indicates the difficulty of validating proposed gene function. Consequently, CoMetGeNe correctly identifies the only reaction in the trail in Fig. 3a that is associated to an annotated gene in *R. baltica* (rba).

*O. terrae* (ote), a member of the subdivision 4 of the Verrucomicrobia phylum, had been thought to be one of the very few exceptions of free-living bacteria without peptidoglycan [45]. Using CoMetGeNe, we however determined that all reactions in the trail in Fig. 3a are present in *O. terrae* (Fig. 3d), with the exception of reaction R03193 which should be catalyzed by MurC. Furthermore, the five present reactions are catalyzed by products of neighboring genes. These CoMetGeNe results are in agreement with the data obtained by Rast et al. [46], who have recently challenged the concept of free-living bacteria lacking peptidoglycan. They proved that members of the Opitutaceae family do possess peptidoglycan cell walls. We propose the candidate *murC* gene in *O. terrae* to be *Oter_2637*, following a protein BLAST [39] for the *E. coli* MurC query sequence (WP_012375453 with 29% identity, 94% query cover, E-value 5e − 41).

### Uncovering unexpected gene ordering patterns

**Fig. 4:**
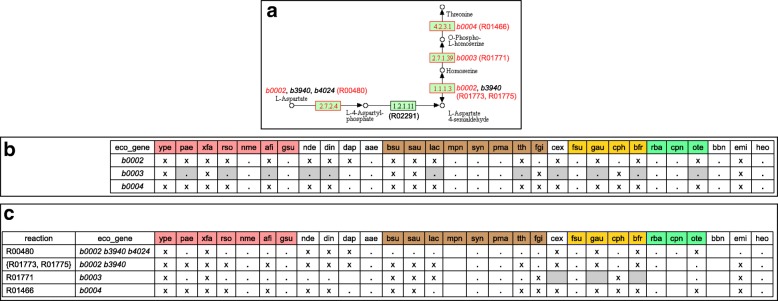
CoMetGeNe trail for *E. coli* in the glycine, serine, and threonine metabolism pathway. **a** Partial view of the glycine, serine, and threonine metabolism pathway, adapted from KEGG PATHWAY, map eco00260 (October 26, 2017 version). Reaction R02291 performing the enzymatic activity 1.2.1.11 was skipped (*δ*_*D*_=1). Genes with black identifiers do not belong to the gene group in **b**. Genes with red identifiers are neighbors on the positive strand of the *E. coli* chromosome. See Fig. 3a for other explanations. **b** Group of homologous genes involved in the trail in **a**, present in various species. Eleven of the species in the dataset (highlighted in gray) either do not have functionally similar genes to *b0003*, or are not contiguous with genes functionally similar to *b0002* and *b0004*. *α*-, *β*-, *γ*-, and *δ*-proteobacteria are highlighted in pink; Terrabacteria, in brown; Sphingobacteria (FCB bacteria), in yellow; and Planctobacteria (PVC bacteria), in light green. **c** Group of reactions defining the trail in **a**. The cells highlighted in gray correspond to the three species among the ones highlighted in gray in **b** that do not perform reaction R01771 (catalyzed by the product of gene *b0003* in *E. coli*)

Figure 4a shows a CoMetGeNe trail for *E. coli* in the glycine, serine, and threonine metabolism pathway (eco00260), representing the conversion of aspartate into threonine. CoMetGeNe produced this trail by skipping reaction R02291 (EC 1.2.1.11), with gap parameter *δ*_*D*_ set to 1.

**Fig. 5:**
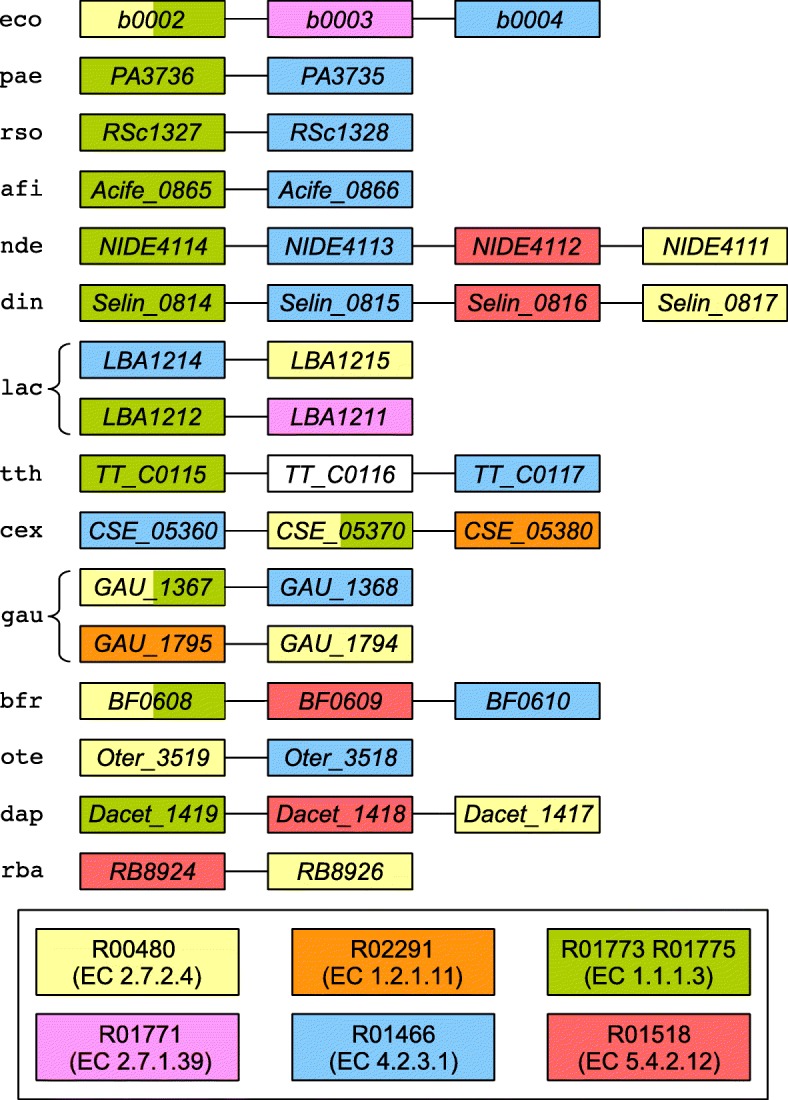
Genomic context for genes involved in the trail in Fig. 4a. Two additional reactions are shown: R02291 (EC 1.2.1.11) linking reactions R00480 (EC 2.7.2.4) and {R01773, R01775} (EC 1.1.1.3), and R01518 (EC 5.4.2.12) representing a phosphoglycerate mutase activity farther along the glycine, serine, and threonine metabolism pathway. Neighboring genes are linked by an edge. Genes are color-coded according to the reactions in which the enzymes they encode take part. Two pairs of neighboring genes on different strands of the bacterial chromosome are shown for *L. acidophilus* (lac) and *G. aurantiaca* (gau). The gene in white in *T. thermophilus* (tth) encodes a hypothetical protein. *D. acetiphilus* (dap) and *R. baltica* (rba) exhibit a similar gene ordering pattern to *N. defluvii* (nde), *D. indicum* (din), and *B. fragilis* (bfr) (see text)

Figures 4b and 4c respectively show the corresponding grouping by genes and by reactions for *E. coli* as reference species and 30 other bacteria from the dataset (trail grouping for the full dataset is available in Additional files 12 and 13). In the case of the 11 species highlighted in gray in Fig. 4b, functionally similar genes to *b0003* are not neighbors of functionally similar genes to *b0002* and *b0004*. The relevant genomic context for these species and two additional ones, *Denitrovibrio acetiphilus* (dap) and *Rhodopirellula baltica* (rba), is shown in Fig. 5.

Figure 4c shows that, of the species highlighted in gray in Fig. 4b, *Caldisericum exile* (cex), *Gemmatimonas aurantiaca* (gau), and *Bacteroides fragilis* (bfr) do not perform reaction R01771 (EC 2.7.1.39), in which the product of gene *b0003* is involved (species highlighted in gray). Only *Lactobacillus acidophilus* (lac) conserved the functionally similar gene *LBA1211* as a neighbor of the gene performing the reaction {R01773, R01775} (see also Fig. 5). The functionally similar genes to *b0003* for the other species highlighted in gray in Fig. 4b exist, but they are located farther on the bacterial chromosome.

Figure 5 shows that strictly neighboring functionally similar genes involved in reactions {R01773, R01775} (EC 1.1.1.3, in green) and R01466 (EC 4.2.3.1, in blue) are conserved for *Pseudomonas aeruginosa* (pae), *Ralstonia solanacearum* (rso), *Acidithiobacillus ferrivorans* (afi), *Nitrospira defluvii* (nde), and *Desulfurispirillum indicum* (din). Interestingly, bi-functional enzymes catalyzing both reactions R00480 (EC 2.7.2.4, in yellow) and {R01773, R01775} (EC 1.1.1.3, in green) are present for *E. coli* (eco), *C. exile* (cex), *G. aurantiaca* (gau), and *B. fragilis* (bfr).

Intriguingly, in species *N. defluvii* (nde), *D. indicum* (din), and *B. fragilis* (bfr), the genes involved in reactions R00480 (EC 2.7.2.4, in yellow) and R01466 (EC 4.2.3.1, in blue) are separated by a gene whose product is involved in the reaction R01518 (EC 5.4.2.12, in red). The bacterial panel was examined in order to determine whether other species exhibit a similar gene ordering pattern. Only *D. acetiphilus* (dap) and *R. baltica* (rba) have neighboring genes involved in R01518 and other reactions from the trail in Fig. 4a. The common denominator for all five species seems to be that the genes whose products catalyze reactions R01518 (EC 5.4.2.12, in red) and R00480 (EC 2.7.2.4, in yellow) are strict neighbors (Fig. 5). Reaction R01518 makes use of a phosphomutase activity for transferring a phosphate group within the same molecule (phosphoglycerate), whereas R00480 employs a phosphotransferase activity for adding a phosphate group to aspartate using ATP. Although there is no obvious link between the two reactions aside from the transfer of a phosphate group, it could be an instance of genomic hitchhiking [47]. This means that operons sometimes contain functionally unrelated genes that nonetheless share similar expression requirements with the rest of the operon. It is possible that gene *apgM* (encoding the enzyme involved in reaction R01518, in red) benefits from the expression levels of genes involved in the trail in Fig. 4a. At any rate, a physiological and/or biochemical reason for co-expression of *apgM* and the gene involved in R00480 (in yellow) seems to exist, since the two genes are neighbors across the bacterial domain, as reported in the STRING database [42].

## Discussion

To contribute insights into the understanding of the complex architecture of pathways forming the primary metabolism [48, 49] and the relationship between metabolism and genomic context [50, 51], we designed CoMetGeNe, a method for the discovery of metabolic and genomic patterns for one species (trail finding) or for a group of species (trail grouping).

Trail finding identifies trails of reactions catalyzed by products of neighboring genes. Flexibility is allowed in the definition of reaction and gene neighborhoods by authorizing that several reactions and/or genes be skipped. Trail finding is an exact approach using graph reduction and path finding in the line graph of the directed graph modeling a metabolic pathway. Path finding in the line graph of a directed graph yields trails in the given directed graph and is based on path enumeration. Since metabolic and genomic data are required for trail finding, the CoMetGeNe pipeline also handles automatic data retrieval from KEGG. Considering the quantity of metabolic and genomic data to be retrieved and analyzed, as well as the exponential nature of the HNET algorithm due to MaSST and MaSSCoT being NP-hard, the total trail finding run time (including data retrieval) for the selected panel of 50 bacterial species (Table 2) was quite satisfactory, amounting to less than 6 hours. Moreover, CoMetGeNe execution time is linear with respect to the number of species to analyze. Data retrieval and trail finding for the 1545 completely assembled representative bacterial genomes present in NCBI Genomes as of November 2018 [52], for instance, requires approximately 8 days. Additional files 14 and 15 contain trail finding results and statistics, respectively, for 1467 of the 1545 species having an identifiable equivalent in KEGG.

Following trail finding, trail grouping is a second step leading from metabolic and genomic patterns for a single species (trails) to the identification of potentially interesting conserved metabolic and genomic patterns in interspecies comparisons. In order to capture the most relevant conserved patterns across multiple species, it is fundamentally important to go beyond strictly matching patterns by accommodating possible trail variations, such as trail directionality, reaction order, repetition of reactions, as well as different but overlapping sets of reactions and/or neighboring genes. The necessity of incorporating these variations for establishing conserved interspecies patterns requires processing trails as reaction sets during the trail grouping step. Once trail grouping has identified potentially interesting conserved patterns, CoMetGeNe users can proceed to analyze the conserved metabolic and genomic patterns between species on a case-by-case basis. During this third analysis step, reaction sets should be considered in their metabolic context and hence treated yet again as trails.

To provide a powerful and flexible way to analyze CoMetGeNe trails, we propose two methods of trail grouping, respectively termed trail grouping by genes and by reactions.

On the one hand, trail grouping by genes is restricted to genes of the reference species that are involved in reaction sets common to at least another species. This approach has the advantage of keeping together neighboring genes that potentially make up for more than a single trail for the reference species. For example, in the peptidoglycan biosynthesis pathway for *Escherichia coli* (map eco00550), CoMetGeNe identified an additional trail of four reactions made up of *ddl* (R01150 in Fig. 3a), *murF*, *mraY*, and *murG*. The gene *ddlB* is a neighbor of *murC* (right side of Fig. 3b). Trail grouping by genes for *E. coli* as reference species results in the group of genes in Fig. 3b plus the additional gene *ddlB*, whereas trail grouping by reactions delineates two distinct groups of reactions: the one in Fig. 3d defining the trail in Fig. 3a, and the aforementioned group of four reactions.

On the other hand, trail grouping by reactions identifies all reaction sets for the reference species, which makes it possible to retrieve valuable information in the form of alternative reactions that might have been filtered out when grouping trails by genes. For example, suppose the reference species is the only species in the panel of species under study to perform a given metabolic route *M*, while also sharing some reactions with other species in the panel. If the shared reactions as well as those specific to the metabolic route *M* involve neighboring genes in the reference species, then the specific route *M*, while not visible when grouping trails by genes, will be present in trail grouping by reactions.

We chose to focus on prokaryotes because of their propensity for organization of genes into operons [53]. Although eukaryotes exhibit gene clustering to a certain extent [54], such an organization is quite infrequent. Additional file 16 contains statistics on genome size, number and percentage of enzyme-coding genes, and CoMetGeNe trails obtained for five eukaryotic species (budding and fission yeast, nematode, zebrafish, and mouse). Overall, we detected fewer trails for eukaryotes by an order of magnitude with respect to bacteria. In terms of span, the median trail span for eukaryotes is approximately half the median trail span in bacteria (see Additional files 6 and 15). Genome fragmentation in the case of eukaryotes with respect to bacteria accounts for the differences in trail detection. Whereas the organization of prokaryotic genes into operons has long been known and studied, CoMetGeNe does not focus specifically on operons. It uncovers them if the resulting proteins are involved in consecutive steps in a metabolic pathway, but it also uncovers genes that are adjacent to operons if the proteins they encode belong to the same trail of reactions. For example, CoMetGeNe identifies a trail of six reactions for *E. coli* in the valine, leucine, and isoleucine biosynthesis pathway (eco00290 in KEGG) representing the conversion of threonine into leucine (data not shown). This trail involves five genes of *E. coli*, four of which constitute the *ilvMEDA* region of the *ilvLGMEDA* operon. The fifth gene, *ilvC*, is not part of this operon as its transcription is regulated by expression of *ilvY* [55].

A further advantage of CoMetGeNe is to disclose missing reactions in various species when grouping trails by reactions. Some instances of missing reactions may indicate the existence of alternative metabolic routes with respect to the reference species, as is the case for *S. aureus*. In other cases, missing reactions suggest that annotations in public knowledge bases may be incorrect, incomplete and/or outdated. We identified a case of incorrect annotation in KEGG for *murF* in *G. sulfurreducens* and we proposed a likely candidate for *murC* in *O. terrae*. We also hypothesize that an error occurred for the prediction of the open reading frame of *ddl* in *F. ginsengisoli*, leading to *ddl* including a domain that may belong to its neighboring gene. If this hypothesis is verified, the redefined coding sequence neighboring *ddl* is likely *murC*.

Another example occurs in the glycine, serine, and threonine metabolism pathway and identifies a trail leading from aspartate to threonine (Fig. 4a). Trail grouping shows that neighboring genes are involved in the trail for numerous species in the selected panel, although one of the reactions is either missing or is performed by the product of a distant gene. Closer investigation reveals an unexpected pattern in gene neighborhood for several of the species in the panel (Fig. 5), where a phosphoglycerate mutase is found to neighbor the aspartate kinase involved in the first reaction in the trail. The pattern is conserved across the bacterial domain, although its biochemical rationale is not readily apparent.

## Conclusions

CoMetGeNe is an exploratory tool determining neighborhood patterns in the metabolic and genomic context of a given species, as well as conserved metabolic and genomic neighborhoods across multiple species. CoMetGeNe may help provide insight into metabolic evolution and reveals the existence of surprising motifs of gene organization. The open-source CoMetGeNe pipeline implementing our method is available at https://cometgene.lri.fr.

## Additional files

Additional file 1 NP-hardness proof. We prove that MaSST and MaSSCoT are NP-hard. (PDF 156 kb)

Additional file 2 Graph reduction proof. Both MaSST and MaSSCoT can take as input graphs *D* and *G* reduced to their respective cover sets of an arc (*u*,*v*) in *D*. We prove that the solution is the same as for *D* and *G* unreduced. (PDF 169 kb)

Additional file 3 CoMetGeNe usage. User manual for the CoMetGeNe pipeline. (PDF 101 kb)

Additional file 4 Trail finding results for the 50 species in Table 2. Trail finding has been performed for all combinations of gap parameters *δ*_*G*_ (allowing to skip genes) and *δ*_*D*_ (allowing to skip reactions) ranging from 0 to 3. (ZIP 5466 kb)

Additional file 5 Input pathways. Metabolic pathways for the selected bacterial species in KGML format, archived. All pathways were retrieved from KEGG June 1, 2018. (ZIP 199,117 kb)

Additional file 6 Statistics per species for the 50 species in Table 2. The included statistics are the total number of genes, the number and percentage of enzyme-coding genes, the number of pathways, the number of trails obtained with CoMetGeNe, average and median trail span, the number of trails of span 1–3, the number of trails of span 4–10, and the number of trails of span 11 or higher. (XLS 20 kb)

Additional file 7 List of 35 reactions in a trail obtained for *Streptococcus pneumoniae* (snd) in the fatty acid biosynthesis pathway (snd00061). The list includes the R numbers of the reactions, the reaction definitions, and the corresponding genes in *S. pneumoniae*. (XLS 12 kb)

Additional file 8 Execution times for the 50 bacterial species in Table 2. For each of the 50 species, the total running time is indicated as well as run times for all combinations of gap parameters *δ*_*G*_ and *δ*_*D*_ ranging from 0 to 3. (XLS 27 kb)

Additional file 9 Comparison between CoMetGeNe and C3Part/Isofun. Both programs were executed on the genome and metabolic pathways of *Escherichia coli*, without skipping any genes or reactions. (ZIP 2853 kb)

Additional file 10 Trail grouping by genes. Group of homologous genes involved in the trail in Fig. 3a (peptidoglycan biosynthesis pathway, eco00550). The reference species is *E. coli* (eco). *α*-, *β*-, *γ*-, and *δ*-proteobacteria are highlighted in pink; Terrabacteria, in brown; Sphingobacteria (FCB bacteria), in yellow; and Planctobacteria (PVC bacteria), in light green. (PDF 20 kb)

Additional file 11 Trail grouping by reactions. Group of reactions defining the trail in Fig. 3a (peptidoglycan biosynthesis pathway, eco00550). The reference species is *E. coli* (eco). For colors used in this figure, see Additional file 10 above. (PDF 21 kb)

Additional file 12 Trail grouping by genes. Group of homologous genes involved in the trail in Fig. 4a (glycine, serine, and threonine metabolism pathway, eco00260). The reference species is *E. coli* (eco). For colors used in this figure, see Additional file 10 above. (PDF 20 kb)

Additional file 13 Trail grouping by reactions. Group of reactions defining the trail in Fig. 4a (glycine, serine, and threonine metabolism pathway, eco00260). The reference species is *E. coli* (eco). For colors used in this figure, see Additional file 10 above. (PDF 21 kb)

Additional file 14 Trail finding results for completely assembled representative bacterial genomes present in NCBI Genomes. Of the 1,545 fully assembled representative bacterial genomes in NCBI, corresponding entries have been identified in KEGG for 1,467 species, for which trail finding has been performed for all combinations of gap parameters *δ*_*G*_ and *δ*_*D*_ ranging from 0 to 3. This additional file is available from https://doi.org/10.6084/m9.figshare.7288769. (GZ 178,532 kb)

Additional file 15 Statistics per species for completely assembled representative bacterial genomes present in NCBI Genomes. The included statistics are the same as for Additional file 6. (XLS 238 kb)

Additional file 16 Statistics per species for five eukaryotic species. Trail finding was conducted on *Saccharomyces cerevisiae*, *Schizosaccharomyces pombe*, *Caenorhabditis elegans*, *Danio rerio*, and *Mus musculus*, with gap parameters *δ*_*G*_ and *δ*_*D*_ ranging from 0 to 3. The included statistics are the same as for Additional file 6. (XLS 7 kb)

## Abbreviations

DAG: Directed acyclic graph
PPI: Protein-protein interaction
SCC: Strongly connected component

## References

[1] Muto A, Kotera M, Tokimatsu T, Nakagawa Z, Goto S, Kanehisa M (2013). Modular architecture of metabolic pathways revealed by conserved sequences of reactions. J Chem Inf Model.

[2] Kanehisa M (2013). Chemical and genomic evolution of enzyme-catalyzed reaction networks. FEBS Lett.

[3] Alves R, Chaleil RA, Sternberg MJ (2002). Evolution of enzymes in metabolism: a network perspective. J Mol Biol.

[4] Rison SC, Teichmann SA, Thornton JM (2002). Homology, pathway distance and chromosomal localization of the small molecule metabolism enzymes in *Escherichia coli*. J Mol Biol.

[5] Zaslaver A, Mayo A, Ronen M, Alon U (2006). Optimal gene partition into operons correlates with gene functional order. Phys Biol.

[6] Wells JN, Bergendahl LT, Marsh JA (2016). Operon gene order is optimized for ordered protein complex assembly. Cell Rep.

[7] Ebrahim A, Brunk E, Tan J, O’brien EJ, Kim D, Szubin R (2016). Multi-omic data integration enables discovery of hidden biological regularities. Nat Commun.

[8] Tohsato Y, Nishimura Y (2008). Metabolic pathway alignment based on similarity between chemical structures. Information and Media Technologies.

[9] Mano A, Tuller T, Béjà O, Pinter RY (2010). Comparative classification of species and the study of pathway evolution based on the alignment of metabolic pathways. BMC Bioinform.

[10] Singh R, Xu J, Berger B (2008). Global alignment of multiple protein interaction networks with application to functional orthology detection. Proc Natl Acad Sci USA.

[11] Neyshabur B, Khadem A, Hashemifar S, Arab SS (2013). NETAL: a new graph-based method for global alignment of protein–protein interaction networks. Bioinformatics.

[12] Laing C, Jung S, Kim N, Elmetwaly S, Zahran M, Schlick T (2013). Predicting helical topologies in RNA junctions as tree graphs. PLoS ONE.

[13] Reinharz V, Soulé A, Westhof E, Waldispühl J, Denise A (2018). Mining for recurrent long-range interactions in RNA structures reveals embedded hierarchies in network families. Nucleic Acids Res.

[14] Chen B, Fan W, Liu J, Wu FX (2013). Identifying protein complexes and functional modules–from static PPI networks to dynamic PPI networks. Brief Bioinform.

[15] Ogata H, Fujibuchi W, Goto S, Kanehisa M (2000). A heuristic graph comparison algorithm and its application to detect functionally related enzyme clusters. Nucleic Acids Res.

[16] Webb EC. Enzyme nomenclature 1992. Recommendations of the Nomenclature Committee of the International Union of Biochemistry and Molecular Biology on the nomenclature and classification of enzymes, Sixth ed.. Academic Press; 1992.

[17] Zheng Y, Szustakowski JD, Fortnow L, Roberts RJ, Kasif S (2002). Computational identification of operons in microbial genomes. Genome Res.

[18] Spirin V, Gelfand MS, Mironov AA, Mirny LA (2006). A metabolic network in the evolutionary context: multiscale structure and modularity. Proc Natl Acad Sci USA.

[19] Boyer F, Morgat A, Labarre L, Pothier J, Viari A (2005). Syntons, metabolons and interactons: an exact graph-theoretical approach for exploring neighbourhood between genomic and functional data. Bioinformatics.

[20] Deniélou YP, Boyer F, Viari A, Sagot MF. Multiple alignment of biological networks: A flexible approach. In: Annual Symposium on Combinatorial Pattern Matching. Springer: 2009. p. 263–273.

[21] Deniélou YP, Sagot MF, Boyer F, Viari A. Bacterial syntenies: an exact approach with gene quorum. 2011; 12(1):193. 10.1186/1471-2105-12-193 PMC3121647 21605461

[22] Bordron P, Eveillard D, Rusu I (2011). Integrated analysis of the gene neighbouring impact on bacterial metabolic networks. IET Systems Biology.

[23] Fertin G, Mohamed-Babou H, Rusu I. Algorithms for subnetwork mining in heterogeneous networks. In: International Symposium on Experimental Algorithms. Springer: 2012. p. 184–194.

[24] Blin G, Fertin G, Mohamed-Babou H, Rusu I, Sikora F, Vialette S. Algorithmic aspects of heterogeneous biological networks comparison. In: International Conference on Combinatorial Optimization and Applications. Springer: 2011. p. 272–286.

[25] Balakrishnan R, Ranganathan K. A textbook of graph theory, 2nd ed. Springer Science & Business Media; 2012.

[26] Kanehisa M, Furumichi M, Tanabe M, Sato Y, Morishima K (2016). KEGG: new perspectives on genomes, pathways, diseases and drugs. Nucleic Acids Res.

[27] Mohamed-Babou H. Comparaison de réseaux biologiques. Ph.D. thesis: Université de Nantes; 2012.

[28] Fertin G, Komusiewicz C, Mohamed-Babou H, Rusu I (2015). Finding supported paths in heterogeneous networks. Algorithms.

[29] Cormen TH, Leiserson CE, Rivest RL, Stein C. Introduction to algorithms, 3rd ed. The MIT Press; 2009.

[30] KEGG API. 2018. http://www.kegg.jp/kegg/rest/keggapi.html. Accessed 7 June 2018.

[31] KEGG organisms: complete genomes. 2018. http://www.kegg.jp/kegg/catalog/org_list.html. Accessed 7 June 2018.

[32] C, 3Part/Isofun. 2018. http://www.inrialpes.fr/helix/people/viari/lxgraph. Accessed 2 Nov 2018.

[33] Boyle DS, Khattar MM, Addinall SG, Lutkenhaus J, Donachie WD (1997). *ftsW*, is an essential cell-division gene in Escherichia coli. Mol Microbiol.

[34] Mohammadi T, Van Dam V, Sijbrandi R, Vernet T, Zapun A, Bouhss A (2011). Identification of FtsW as a transporter of lipid-linked cell wall precursors across the membrane. EMBO J.

[35] Waites KB, Talkington DF (2004). *Mycoplasma pneumoniae*, and its role as a human pathogen. Clin Microbiol Rev.

[36] Caccavo F, Lonergan DJ, Lovley DR, Davis M, Stolz JF, McInerney MJ (1994). *Geobacter sulfurreducens* sp. nov., a hydrogen- and acetate-oxidizing dissimilatory metal-reducing microorganism. Appl Environ Microbiol.

[37] Mahadevan R, Bond DR, Butler JE, Coppi MV, Palsson BO, Esteve-Núñez A (2006). Characterization of metabolism in the Fe(III)-reducing organism *Geobacter sulfurreducens* by constraint-based modeling. Appl Environ Microbiol.

[38] KEGG GENES entry for GSU3073 (*Geobacter sulfurreducens* PCA). 2018. http://www.genome.jp/dbget-bin/www_bget?gsu:GSU3073. Accessed 7 June 2018.

[39] Altschul SF, Madden TL, Zhang J, Zhang Z, Miller W, Schäffer AA (1997). Gapped BLAST and PSI-BLAST: a new generation of protein database search programs. Nucleic Acids Res.

[40] Willey JM, Sherwood LM, Woolverton CJ. Bacteria: the low G+C Gram positives. In: Prescott, Harley, and Klein’s Microbiology, 7th Ed. McGraw-Hill Higher Education: 2008. p. 571–588.

[41] Im WT, Hu ZY, Kim KH, Rhee SK, Meng H, Lee ST (2012). Description of *Fimbriimonas ginsengisoli*, gen. nov., sp. nov. within the *Fimbriimonadia*, class nov., of the phylum *Armatimonadetes*. Antonie Van Leeuwenhoek.

[42] Szklarczyk D, Franceschini A, Wyder S, Forslund K, Heller D, Huerta-Cepas J (2014). STRING v10: protein–protein interaction networks, integrated over the tree of life. Nucleic Acids Res.

[43] Fuerst JA, Sagulenko E (2011). Beyond the bacterium: planctomycetes challenge our concepts of microbial structure and function. Nat Rev Microbiol.

[44] Jeske O, Schumann P, Schneider A, Boedeker C, Jogler M, Schüuler M (2015). Planctomycetes do possess a peptidoglycan cell wall. Nat Commun.

[45] Yoon J (2011). Phylogenetic studies on the bacterial phylum Verrucomicrobia. Microbiol Cult Coll.

[46] Rast P, Glöockner I, Boedeker C, Jeske O, Wiegand S, Reinhardt R (2017). Three novel species with peptidoglycan cell walls form the new genus *Lacunisphaera*, gen. nov. in the family Opitutaceae of the verrucomicrobial subdivision 4. Frontiers in Microbiology.

[47] Rogozin IB, Makarova KS, Murvai J, Czabarka E, Wolf YI, Tatusov RL (2002). Connected gene neighborhoods in prokaryotic genomes. Nucleic Acids Res.

[48] Jeong H, Tombor B, Albert R, Oltvai ZN, Barabási AL (2000). The large-scale organization of metabolic networks. Nature.

[49] Schmitt DL, An S (2017). Spatial organization of metabolic enzyme complexes in cells. Biochemistry.

[50] Vitkup D, Kharchenko P, Wagner A (2006). Influence of metabolic network structure and function on enzyme evolution. Genome Biol.

[51] Copley SD (2012). Toward a systems biology perspective on enzyme evolution. J Biol Chem.

[52] NCBI genome list. 2018. https://www.ncbi.nlm.nih.gov/genome/browse#!/prokaryotes/. Accessed 1 Nov 2018.

[53] Moreno-Hagelsieb G (2015). The power of operon rearrangements for predicting functional associations. Comput Struct Biotechnol J.

[54] Hurst LD, Lercher MJ, Pál C (2004). The evolutionary dynamics of eukaryotic gene order. Nat Rev Genet.

[55] Wek RC, Hatfield GW (1988). Transcriptional activation at adjacent operators in the divergent-overlapping *ilvY*, and *ilvC*, promoters of *Escherichia coli*. J Mol Biol.

