# Supplementary material for: CoMetGeNe: mining conserved neighborhood patterns in metabolic and genomic contexts
Source: BMC Bioinformatics. 2019 Jan 10;20:19. doi: 10.1186/s12859-018-2542-2 (PMC6327494; doi:10.1186/s12859-018-2542-2)
Supplement: Supplementary file 1 — NP-hardness proof. We prove that MaSST and MaSSCoT are NP-hard. (PDF 156 kb) [file 12859_2018_2542_MOESM1_ESM.pdf]

CoMetGeNe: mining conserved neighborhood patterns in metabolic and genomic contexts

Alexandra Zaharia, Bernard Labedan, Christine Froidevaux, Alain Denise

NP-hardness proof sketch

Longest Supported Path (LSP)
**Input:** A directed graph $D = (V, A)$, an undirected graph $G = (V, E)$.
**Solution:** A longest path P in D such that $G[V(P)]$ is connected.

It is known that LSP is NP-hard for general graphs D and G; It remains NP-hard even if D is acyclic and G is a tree with diameter 4 [1, 2].

Maximum Span Supported Trail (MaSST)
**Input:** A directed graph $D = (V, A)$, an undirected graph $G = (V, E)$, an arc (u, v) in D.
**Solution:** A trail of maximum span T in D passing through (u, v) such that $G[V(T)]$ is connected.

Maximum Span Supported Corresponding Trail (MaSSCoT)
**Input:** A directed graph $D = (V, A)$, an undirected graph $G = (V, E)$, an arc (u, v) in D.
**Solution:** A path P in the line graph of D such that $L^{-1}(P)$ has maximum span, passes through (u, v), and $G[V(L^{-1}(P))]$ is connected.

Let us now state Maximum Span Trail (MaST), a problem formulation closely related to MaSST:

Maximum Span Trail (MaST)
**Input:** A directed graph $D = (V, A)$, an undirected graph $G = (V, E)$.
**Solution:** A trail of maximum span T in D such that $G[V(T)]$ is connected.

Proposition 1 MaST is NP-hard.

Proof. We know that LSP is NP-hard even if D is acyclic. Now, if D is acyclic, then LSP and MaST have exactly the same solution. Thus MaST is NP-hard (even if D is acyclic). □

Proposition 2 (Corollary of proposition 1) MaSST is NP-hard.

Proof. Suppose that MaSST is polynomially tractable. Then, by applying it on all arcs of D in turn, MaST can be solved in polynomial time as well. But MaST is NP-hard (proposition 1). □

Lemma 1 Let $D = (V, A)$ be a directed graph and $L(D) = (A, A')$ be its line graph. Let $P = (a_1,\ a_2,\ \ldots,\ a_k)$ be a path in $L(D)$, where $a_i = (t_{i-1},\ t_i)\ \forall i \in \{1,\ \ldots,\ k\}$ are edges in D. Then the unique sequence of vertices $(t_0,\ t_1,\ t_2,\ \ldots,\ t_{k-1},\ t_k)$ associated to P is a trail in D.

Proof. By construction of P we have that the sequence of vertices $T = (t_0,\ t_1,\ t_2,\ \ldots,\ t_{k-1},\ t_k)$ is unique and is a walk in D. Since P has no repeated vertices, T contains no repeated arcs. T is therefore a trail in D. □

Proposition 3 (Corollary of proposition 2) MaSSCoT is NP-hard.

Proof. A path in the line graph of a directed graph D is a trail in D (lemma 1). Let $T = L^{-1}(P)$ be the trail in D corresponding to P. The solution to MaSSCoT is then a trail of maximum span T in D passing through (u, v) such that $G[V(T)]$ is connected. MaSST and MaSSCoT are therefore equivalent and NP-hard (proposition 2). □

References

[1] Fertin G, Komusiewicz C, Mohamed-Babou H, Rusu I. Finding supported paths in heterogeneous networks. Algorithms. 2015;8(4):810–831.

[2] Fertin G, Mohamed-Babou H, Rusu I. Algorithms for subnetwork mining in heterogeneous networks. In: International Symposium on Experimental Algorithms. Springer; 2012. p. 184–194.
