# Supplementary material for: CoMetGeNe: mining conserved neighborhood patterns in metabolic and genomic contexts
Source: BMC Bioinformatics. 2019 Jan 10;20:19. doi: 10.1186/s12859-018-2542-2 (PMC6327494; doi:10.1186/s12859-018-2542-2)
Supplement: Supplementary file 2 — Graph reduction proof. Both MaSST and MaSSCoT can take as input graphs D and G reduced to their respective cover sets of an arc (u,v) in D. We prove that the solution is the same as for D and G unreduced. (PDF 169 kb) [file 12859_2018_2542_MOESM2_ESM.pdf]

# CoMetGeNe: mining conserved neighborhood patterns in metabolic and genomic contexts

Alexandra Zaharia, Bernard Labedan, Christine Froidevaux, Alain Denise

## Reduction of $D$ and $G$ to the cover set of an arc of $D$

In this document we prove that reducing the input graphs $D$ and $G$ (for MaSST and MaSSCoT) to their cover set with respect to a path $P$ in $D$ yields the same solution as for the versions without reduction.

Assuming $D$ is a directed graph, the following notations will be used:

- $D^*$ is the underlying undirected graph of $D$ obtained by removing arc orientations.
- If $G$ is an undirected graph such that $V(D) = V(G)$ and $P$ is a path in $D$, the notation $CCC(D^*,\ G,\ P)$ designates the common connected component of $D^*$ and $G$ containing all vertices in $P$, if such a component exists. If it does not, then $CCC(D^*,\ G,\ P) = \varnothing$.
- For a vertex $v$ of $D$, $S_v^+$ designates the descendants of $v$, i.e. the set of vertices in $D$ that are reachable by a path from vertex $v$.
- For a vertex $v$ of $D$, $S_v^-$ designates the ancestors of $v$, i.e. the set of vertices in $D$ reaching vertex $v$ by a path in $D$.

The definition of cover set of a path uses the concept of bridge, defined as follows by Fertin *et al.* [1]:

**Definition 1** Let $D = (V, A)$ be a directed graph, $G = (V, E)$ an undirected graph, and $P$ a path in $D$. A vertex $r \in V$ is said to be a *bridge of P with respect to G* if there is no common connected component of $D^*[V - \{r\}]$ and $G[V - \{r\}]$ containing all the vertices of $P$ (i.e., $CCC(D^*[V - \{r\}],\ G[V - \{r\}],\ P) = \varnothing$).

Fertin *et al.* [1] define the cover set of a path as follows:

**Definition 2** Let $D = (V, A)$ be a directed graph, $G = (V, E)$ an undirected graph, and $P = (v_1,\ v_2,\ \ldots,\ v_k)$ a path in $D$. The *cover set of path P in D with respect to G* is the set $X$ satisfying:

1. $V(P) \subseteq X \subseteq S_{v_1}^- \cup S_{v_k}^+ \cup V(P)$.
2. $D^*[X]$ and $G[X]$ are connected.
3. If $r$ is a bridge of $P$ in $D[X]$ with respect to $G[X]$ then $X \subseteq S_r^- \cup S_r^+ \cup \{r\}$.
4. $X$ is maximal (with respect to the inclusion order).

Both MaSST and MaSSCoT take as input a directed graph $D = (V, A)$, an undirected graph $G = (V, E)$, and an arc $(u, v)$ in $D$. Let $S$ be the cover set of arc $(u, v)$ in $D$ with respect to $G$. We prove that $D$ and $G$ can respectively be replaced with $D[S]$ and $G[S]$, yielding the same solutions.

Following is a lemma for which the proof is omitted because it is trivial.

**Lemma 1** Let $G = (V, E)$ be an undirected graph and let $A$, $B$ and $C$ be three subsets of $V$. If $G[A \cup B]$ and $G[B \cup C]$ are connected, then $G[A \cup B \cup C]$ is connected.

Next, we introduce a definition that will serve as a shorthand notation for the remainder of this document.

**Definition 3** Let $D = (V, A)$ be a directed graph, $G = (V, E)$ be an undirected graph and $P$ be a path in $D$. If a trail $T$ in $D$ exists such that (i) $T \supseteq P$, (ii) $G[V(T)]$ is connected and (iii) $\nexists T'$ trail in $D$ such that $T' \supseteq P$, $G[V(T')]$ is connected and $span(T') > span(T)$, then $T$ is said to be a *trail of maximum span in D for P and G with respect to* (i) *and* (ii), and $T$ is said to *verify* $\mathscr{S}_P(D,\ G)$.

**Lemma 2** Let $D = (V, A)$ be a directed graph, $G = (V, E)$ an undirected graph, $P$ a path in $D$, and $S$ the cover set of $P$ in graphs $D$ and $G$. If a trail $T$ in $D$ exists such that $T$ verifies $\mathscr{S}_P(D,\ G)$, then $V(T) \subseteq S$.

*Proof.* $T \supseteq P$ by hypothesis (i) of definition 3 and $S \supseteq V(P)$ by definition of the cover set. It follows that $V(T) \cap S \neq \varnothing$. Let $I = V(T) \cap S$ be the vertices shared by $T$ and $S$. Let $\{t_1,\ \ldots,\ t_k\} = V(T) - I$ be the vertices of $T$ not shared with $S$.

$G[V(T)]$ is connected by hypothesis (ii) of definition 3, therefore $G[\{t_1,\ \ldots,\ t_k\} \cup I]$ is connected. Since $G[S]$ is connected (by definition of the cover set), it means that $G[\{t_1,\ \ldots,\ t_k\} \cup S] = G[\{t_1,\ \ldots,\ t_k\} \cup I \cup (S - I)]$ is also connected (by lemma 1). Also, $D^*[V(T)]$ is connected because $T$ is a trail in $D$, which means that $D^*[\{t_1,\ \ldots,\ t_k\} \cup I]$ is connected. Since $D^*[S]$ is connected (by definition of the cover set), it means that $D^*[\{t_1,\ \ldots,\ t_k\} \cup S] = D^*[\{t_1,\ \ldots,\ t_k\} \cup I \cup (S - I)]$ is also connected (by lemma 1).

But if $G[\{t_1,\ \ldots,\ t_k\} \cup S]$ and $D^*[\{t_1,\ \ldots,\ t_k\} \cup S]$ are connected, then property 4 of definition 2 is contradicted, therefore $S$ cannot be maximal unless $\{t_1,\ \ldots,\ t_k\} = \varnothing$. Hence $V(T) \subseteq S$. □

**Proposition 1** Let $D = (V, A)$ be a directed graph, $G = (V, E)$ an undirected graph, $P$ a path in $D$, and $S$ the cover set of $P$ in graphs $D$ and $G$. If a trail $T$ in $D$ exists such that $T$ verifies $\mathscr{S}_P(D,\ G)$, then $T$ is also a trail in $D[S]$ verifying $\mathscr{S}_P(D[S],\ G[S])$, that is, (i) $T \supseteq P$, (ii) $G[S \cap V(T)]$ is connected and (iii) $T$ has maximum span in $D[S]$ with respect to (i) and (ii).

*Proof.* Let $T$ be a trail in $D$ such that $T$ verifies $\mathscr{S}_P(D,\ G)$. By lemma 2, $V(T) \subseteq S$, therefore $T$ is also a trail in $D[S]$. We will now prove that $T$ verifies properties (i)-(iii) for graphs $D[S]$ and $G[S]$.

(i) By hypothesis (i) of definition 3, $T \supseteq P$.

(ii) Since $V(T) \subseteq S$, $G[S \cap V(T)] = G[V(T)]$. Since $G[V(T)]$ is connected by hypothesis (ii) of definition 3, it follows that $G[S \cap V(T)]$ is connected.

(iii) Suppose there exists a trail $T'$ in $D[S]$ verifying $\mathscr{S}_P(D[S],\ G[S])$, that is, $T' \supseteq P$, $G[S \cap V(T')]$ is connected and $span(T') > span(T)$. Because $T'$ is a trail in $D[S]$, $V(T') \subseteq S$, which implies that $S \cap V(T') = V(T')$. Since $G[S \cap V(T')]$ is connected, it follows that $G[V(T')]$ is also connected. Moreover, $S \subseteq V$ by definition 2, therefore $T'$ is also a trail in $D$. Hypotheses (i)-(iii) of definition 3 are thus fulfilled for $T'$ in $D$, meaning that $T'$ verifies $\mathscr{S}_P(D,\ G)$, which contradicts the fact that $T$ verifies $\mathscr{S}_P(D,\ G)$. Hence, no trail $T'$ can exist in $D[S]$ that includes $P$ such that $G[S \cap V(T')]$ is connected and such that $T'$ has greater span than $T$. $T$ has therefore maximum span in $D[S]$ with respect to properties (i) and (ii).

We have proved that, if a trail $T$ in $D$ exists such that $T$ verifies $\mathscr{S}_P(D,\ G)$, then $T$ is also a trail in $D[S]$ that verifies $\mathscr{S}_P(D[S],\ G[S])$. □

**Proposition 2** Let $D = (V, A)$ be a directed graph, $G = (V, E)$ an undirected graph, $P$ a path in $D$, and $S$ the cover set of $P$ in graphs $D$ and $G$. If a trail $T$ in $D[S]$ exists that verifies $\mathscr{S}_P(D[S],\ G[S])$, then $T$ is also a trail in $D$ verifying $\mathscr{S}_P(D,\ G)$, that is, (i) $T \supseteq P$, (ii) $G[V(T)]$ is connected and (iii) $T$ has maximum span in $D$ with respect to (i) and (ii).

*Proof.* This proposition is the converse of proposition 1. Let $T$ be a trail in $D[S]$ such that $T$ verifies $\mathscr{S}_P(D[S],\ G[S])$. By definition 2, $S \subseteq V$, therefore $T$ is also a trail in $D$. We will now prove that $T$ verifies properties (i)-(iii) for graphs $D$ and $G$.

(i) By hypothesis (i) of definition 3, $T \supseteq P$.

(ii) Since $T$ is a trail in $D[S]$, $V(T) \subseteq S$. Then $S \cap V(T) = V(T)$, and since $G[S \cap V(T)]$ is connected by hypothesis (ii) of definition 3, it follows that $G[V(T)]$ is connected.

(iii) Suppose there exists a trail $T'$ in $D$ verifying $\mathscr{S}_P(D,\ G)$, that is, $T' \supseteq P$, $G[V(T')]$ is connected and $span(T') > span(T)$. By lemma 2, $V(T') \subseteq S$ and therefore $S \cap V(T') = V(T')$. Since $G[V(T')]$ is connected, it follows that $G[S \cap V(T')]$ is also connected. Moreover, $T'$ is also a trail in $D[S]$

(because $V(T') \subseteq S$). Hypotheses (i)-(iii) of definition 3 are thus fulfilled for $T'$ in $D[S]$, meaning that $T'$ verifies $\mathscr{S}_P(D[S],\ G[S])$, which contradicts the fact that $T$ verifies $\mathscr{S}_P(D[S],\ G[S])$. Hence, no trail $T'$ can exist in $D$ that includes $P$ such that $G[V(T')]$ is connected and such that $T'$ has greater span than $T$. $T$ has therefore maximum span in $D$ with respect to properties (i) and (ii).

We have proved that, if a trail $T$ in $D[S]$ exists such that $T$ verifies $\mathscr{S}_P(D[S],\ G[S])$, then $T$ is also a trail in $D$ that verifies $\mathscr{S}_P(D,\ G)$. □

# References


[1] Fertin G, Mohamed-Babou H, Rusu I. Algorithms for subnetwork mining in heterogeneous networks. In: International Symposium on Experimental Algorithms. Springer; 2012. p. 184–194.
