# Supplementary material for: CoMetGeNe: mining conserved neighborhood patterns in metabolic and genomic contexts
Source: BMC Bioinformatics. 2019 Jan 10;20:19. doi: 10.1186/s12859-018-2542-2 (PMC6327494; doi:10.1186/s12859-018-2542-2)
Supplement: Supplementary file 3 — CoMetGeNe usage. User manual for the CoMetGeNe pipeline. (PDF 101 kb) [file 12859_2018_2542_MOESM3_ESM.pdf]

CoMetGeNe: mining conserved neighborhood patterns in metabolic and genomic contexts

Alexandra Zaharia, Bernard Labedan, Christine Froidevaux, Alain Denise

`CoMetGeNe` usage

`CoMetGeNe.py`

Suppose `CoMetGeNe` is executed as follows: `CoMetGeNe.py eco data/ -dG 2 -dD 1 -o eco.out`.

Metabolic pathways for *Escherichia coli* K-12 MG1655 (`eco`) are automatically downloaded from KEGG and stored under `data/`. At most two genes (`-dG 2`) and one reaction (`-dD 1`) can be skipped. Results are saved in the output file `eco.out`.

Detailed `CoMetGeNe.py` options follow:

```
usage: CoMetGeNe.py [-h] [--delta_G NUMBER] [--delta_D NUMBER] [--timeout SECONDS]
                    [--output OUTPUT] [--skip-import]
                    ORG DIR

Determines maximum trails of reactions for the specified organisms such that the
genes coding for the enzymes involved in the trail are neighbors.

A trail of reactions is a sequence of reactions that can repeat reactions
(vertices), but not arcs between reactions.

Metabolic pathways and genomic information is automatically retrieved from the
KEGG database.


Required arguments:
  ORG                   query organism (three- or four-letter KEGG code, e.g.
                        'eco' for Escherichia coli K-12 MG1655). See full
                        list of KEGG organism codes at
                        http://rest.kegg.jp/list/genome
  DIR                   directory storing metabolic pathways for the query
                        organism ORG or where metabolic pathways for ORG will
                        be downloaded

Optional arguments:
  -h, --help            show this help message and exit
  --delta_G NUMBER, -dG NUMBER
                        the NUMBER of genes that can be skipped (default: 0)
  --delta_D NUMBER, -dD NUMBER
                        the NUMBER of reactions that can be skipped (default:
                        0)
  --timeout SECONDS, -t SECONDS
                        timeout in SECONDS (default: 300)
  --output OUTPUT, -o OUTPUT
                        output file
  --skip-import, -s     skips importing metabolic pathways from KEGG,
```

```
                        attempting to use locally stored KGML files if they
                        are present under the specified directory (DIR)

Example: running

    python2 CoMetGeNe.py eco data/ -dG 2 -o eco.out

downloads metabolic pathways for species 'eco' to directory 'data/'. Trail
finding is performed, allowing two genes to be skipped at most (-dG 2).
Reactions cannot be skipped (-dD is 0 by default). Maximum trails of reactions
such that the reactions are catalyzed by products of neighboring genes are saved
in the output file 'eco.out'.
```

grouping.py

For example, suppose `grouping` is executed as follows: `grouping.py genes results/ data/ eco -o tsg_eco.csv`.

Trail grouping by `genes` is performed for species `eco` and the resulting table $T^g_{\texttt{eco}}$ is saved in `tsg_eco.csv`. `CoMetGeNe` results for all species and metabolic pathways of `eco` are respectively assumed to exist in `results/` and `data/`.

Detailed `grouping.py` options follow:

```
usage: grouping.py [-h] [--output OUTPUT] {genes,reactions} RESULTS KGML ORG

Groups CoMetGeNe trails by either genes or reactions, producing a CSV file.

Required arguments:
  {genes,reactions}     type of trail grouping to perform (possible values:
                        'genes' or 'reactions')
  RESULTS               directory storing CoMetGeNe results
  KGML                  directory containing input KGML files
  ORG                   reference species (KEGG organism code)

Optional arguments:
  -h, --help            show this help message and exit
  --output OUTPUT, -o OUTPUT
                        output file (CSV)

KGML needs to contain a subdirectory for every species for which a result file
is present in RESULTS. The subdirectory names need to be the three- or four-
letter KEGG codes for the species in question (e.g., 'bsu', 'eco', 'pae',
etc.). Each species subdirectory is expected to contain metabolic pathways in
KGML format.

Example: running

    python2 grouping.py genes results/ data/ eco -o grouping_gene_eco.csv

will perform trail grouping by genes for the reference species 'eco'. The
CoMetGeNe results are stored in 'results/', and the KGML files are available in
'data/'. A CSV file is produced ('grouping_gene_eco.csv').
```
