# Supplementary material for: CoMetGeNe: mining conserved neighborhood patterns in metabolic and genomic contexts
Source: BMC Bioinformatics. 2019 Jan 10;20:19. doi: 10.1186/s12859-018-2542-2 (PMC6327494; doi:10.1186/s12859-018-2542-2)
Supplement: Supplementary file 10 — Trail grouping by genes. Group of homologous genes involved in the trail in Fig. 3a (peptidoglycan biosynthesis pathway, eco00550). The reference species is E. coli (eco). α-, β-, γ-, and δ-proteobacteria are highlighted in pink; Terrabacteria, in brown; Sphingobacteria (FCB bacteria), in yellow; and Planctobacteria (PVC bacteria), in light green. (PDF 20 kb) [file 12859_2018_2542_MOESM10_ESM.pdf]

eco00550_genes

eco_gene	str	pathway	ype	vco	spc	pae	xfa	rso	nme	afi	ara	rrj	gsu	nde	aca	din	fnu	dap	tid	aae	bsu	lmo	sau	lac	snd	cpe	mpn	syn	pma	cau	bbv	cgl	mtv	sco	dra	tth	fgi	amo	tmm	cex	dth	fsu	gau	cph	bfr	rba	cpn	ote	bbn	emi	heo
b0085	+	00550 00300	x	x	x	x	x	x	x	x	x	x	x	x	x	.	.	x	x	.	x	x	.	.	.	x	.	.	.	.	.	x	x	x	.	.	x	x	x	x	x	x	x	x	x	.	.	x	.	.	.
b0086	+	00550 00300	x	x	x	x	x	x	x	x	x	x	.	x	x	x	x	x	x	.	.	.	.	.	.	x	.	.	.	x	x	x	x	x	.	x	x	x	x	x	x	x	x	x	.	.	x	x	x	x	.
b0087	+	00550	x	x	x	x	x	x	x	x	x	x	x	x	x	x	x	x	x	.	x	x	x	x	.	x	.	.	.	x	x	x	x	x	.	x	x	x	x	x	x	.	x	x	x	.	x	x	x	x	x
b0088	+	00550 00471	x	x	x	x	.	x	x	x	x	.	x	x	x	x	x	x	x	.	x	x	x	x	x	.	.	.	.	x	x	x	x	x	.	x	x	x	x	x	x	.	x	x	x	.	x	x	.	.	x
b0090	+	00550	x	x	x	x	x	x	x	x	x	.	x	x	x	x	x	x	x	.	x	x	.	x	x	.	.	.	.	x	x	x	x	x	x	x	x	x	x	x	x	x	x	x	x	.	x	x	.	x	.
b0091	+	00550 00471	x	x	x	x	x	x	x	x	x	.	x	x	x	.	x	x	x	.	.	.	.	.	.	.	.	.	.	x	x	x	x	.	x	x	.	x	x	x	x	x	x	x	x	.	x	.	.	.	.
