# Supplementary material for: CoMetGeNe: mining conserved neighborhood patterns in metabolic and genomic contexts
Source: BMC Bioinformatics. 2019 Jan 10;20:19. doi: 10.1186/s12859-018-2542-2 (PMC6327494; doi:10.1186/s12859-018-2542-2)
Supplement: Supplementary file 11 — Trail grouping by reactions. Group of reactions defining the trail in Fig. 3a (peptidoglycan biosynthesis pathway, eco00550). The reference species is E. coli (eco). For colors used in this figure, see Additional file 10 above. (PDF 21 kb) [file 12859_2018_2542_MOESM11_ESM.pdf]

eco00550_reactions

reaction	eco_gene	pathway	ype	vco	spc	pae	xfa	rso	nme	afi	ara	rrj	gsu	nde	aca	din	fnu	dap	tid	aae	bsu	lmo	sau	lac	snd	cpe	mpn	syn	pma	cau	bbv	cgl	mtv	sco	dra	tth	fgi	amo	tmm	cex	dth	fsu	gau	cph	bfr	rba	cpn	ote	bbn	emi	heo
R03193	b0091	00550 00471	x	x	x	x	x	x	x	x	x	.	x	x	x	.	x	x	x	.	.	.	.	.	.	.		.	.	x	x	x	x	.	x	x		x	x	x	x	x	x	x	x		x		.	.	.
R02783	b0088	00550 00471	x	x	x	x	.	x	x	x	x	.	x	x	x	x	x	x	x	.	x	x	x	x	x	.		.	.	x	x	x	x	x	.	x	x	x	x	x	x	.	x	x	x		x	x	.	.	x
R02788	b0085	00550 00300	x	x	x	x	x	x	x	x	x	x	x	x	x	.	.	x	x	.	x	x		.	.	x		.	.	.	.	x	x	x	.	.	x	x	x	x	x	x	x	x	x	.	.	x	.	.	.
R04617	b0086	00550 00300	x	x	x	x	x	x	x	x	x	x		x	x	x	x	x	x	.	.	.	.	.	.	x		.	.	x	x	x	x	x	.	x	x	x	x	x	x	x	x	x	.		x	x	x	x	.
R05630	b0087	00550	x	x	x	x	x	x	x	x	x	x	x	x	x	x	x	x	x	.	x	x	x	x	.	x		.	.	x	x	x	x	x	.	x	x	x	x	x	x	.	x	x	x		x	x	x	x	x
R05032	b0090	00550	x	x	x	x	x	x	x	x	x	.	x	x	x	x	x	x	x	.	x	x	.	x	x	.		.	.	x	x	x	x	x	x	x	x	x	x	x	x	x	x	x	x		x	x	.	x	.
