# Supplementary material for: CoMetGeNe: mining conserved neighborhood patterns in metabolic and genomic contexts
Source: BMC Bioinformatics. 2019 Jan 10;20:19. doi: 10.1186/s12859-018-2542-2 (PMC6327494; doi:10.1186/s12859-018-2542-2)
Supplement: Supplementary file 12 — Trail grouping by genes. Group of homologous genes involved in the trail in Fig. 4a (glycine, serine, and threonine metabolism pathway, eco00260). The reference species is E. coli (eco). For colors used in this figure, see Additional file 10 above. (PDF 20 kb) [file 12859_2018_2542_MOESM12_ESM.pdf]

eco00260_genes

eco_gene	str	pathway	ype	vco	spc	pae	xfa	rso	nme	afi	ara	rrj	gsu	nde	aca	din	fnu	dap	tid	aae	bsu	lmo	sau	lac	snd	cpe	mpn	syn	pma	cau	bbv	cgl	mtv	sco	dra	tth	fgi	amo	tmm	cex	dth	fsu	gau	cph	bfr	rba	cpn	ote	bbn	emi	heo
b0002	+	00260 00261 00270 00300	x	x	x	x	x	x	.	x	.	.	.	x	.	x	.	x	.	.	x	x	x	x	x	.	.	.	.	.	x	x	x	x	.	x	.	x	x	x	x	.	x	.	x	.	.	x	.	x	.
b0003	+	00260	x	x	x	.	x	.	.	.	.	.	.	.	x	.	.	.	.	.	x	x	x	.	x	.	.	.	.	.	x	x	x	x	.	.	x	x	x	.	x	.	.	x	.	.	.	.	.	x	.
b0004	+	00260 00750	x	x	x	x	x	x	.	x	.	.	.	x	x	x	.	.	.	.	x	x	x	x	.	.	.	.	.	.	.	.	x	x	.	x	x	x	x	x	x	.	x	x	x	.	.	x	.	x	.
