# Supplementary material for: CoMetGeNe: mining conserved neighborhood patterns in metabolic and genomic contexts
Source: BMC Bioinformatics. 2019 Jan 10;20:19. doi: 10.1186/s12859-018-2542-2 (PMC6327494; doi:10.1186/s12859-018-2542-2)
Supplement: Supplementary file 13 — Trail grouping by reactions. Group of reactions defining the trail in Fig. 4a (glycine, serine, and threonine metabolism pathway, eco00260). The reference species is E. coli (eco). For colors used in this figure, see Additional file 10 above. (PDF 21 kb) [file 12859_2018_2542_MOESM13_ESM.pdf]

# eco00260_reactions

| reaction | eco_gene | pathway | ype | vco | spc | pae | xfa | rso | nme | afi | ara | rrj | gsu | nde | aca | din | fnu | dap | tid | aae | bsu | lmo | sau | lac | snd | cpe | mpn | syn | pma | cau | bbv | cgl | mtv | sco | dra | tth | fgi | amo | tmm | cex | dth | fsu | gau | cph | bfr | rba | cpn | ote | bbn | emi | heo |
|---|---|---|---|---|---|---|---|---|---|---|---|---|---|---|---|---|---|---|---|---|---|---|---|---|---|---|---|---|---|---|---|---|---|---|---|---|---|---|---|---|---|---|---|---|---|---|---|---|---|---|---|---|---|---|
| R00480 | b4024 b0002 b3940 | 00260 00261 00270 00300 | x | x | x | . | x | . | . | . | . | . | . | x | . | x | | x | . | . | . | . | . | . | . | . | | . | . | . | | . | . | . | . | . | . | x | x | x | x | . | x | . | x | . | . | x | | . | . |
| {R01773, R01775} | b0002 b3940 | 00260 00270 00300 | x | x | x | x | x | x | . | x | . | | . | x | . | x | | x | . | . | x | x | x | x | x | | | . | . | . | x | x | x | x | . | x | . | . | x | x | x | . | x | . | x | . | | . | | x | . |
| R01771 | b0003 | 00260 | x | x | x | . | x | . | . | . | . | | | . | x | . | . | | | . | x | x | x | x | x | | | . | . | . | x | x | x | x | . | . | x | x | x | | x | . | | x | | | | . | | x | . |
| R01466 | b0004 | 00260 | x | x | x | x | x | x | . | x | . | | . | x | x | x | | . | . | . | x | x | x | . | . | | | . | . | . | . | . | x | x | . | x | x | x | x | x | x | . | x | x | x | . | | x | | x | . |
